# Self-resistance mechanism to acyldepsipeptide antibiotics in the *Streptomyces* producer

**DOI:** 10.1128/mbio.01652-25

**Published:** 2025-10-06

**Authors:** Dhana Thomy, Laura Reinhardt, Elisa Liebhart, Mirita Franz-Wachtel, Boris Maček, Peter Sass, Heike Brötz-Oesterhelt

**Affiliations:** 1 Department of Microbial Bioactive Compounds, Interfaculty Institute of Microbiology and Infection Medicine, University of Tübingen, Tübingen, Germany; 2 Cluster of Excellence—Controlling Microbes to Fight Infections, University of Tübingen 9188, Tübingen, Germany; 3 Proteome Center Tübingen, University of Tübingen 9188, Tübingen, Germany; Ludwig-Maximilians-Universität München, Munich, Germany

**Keywords:** ADEP, *Streptomyces hawaiiensis*, natural products, producer self-protection, ClpP, Clp protease

## Abstract

Clp proteases are ubiquitous in bacteria and play an important role in regulatory proteolysis and in maintaining protein homeostasis within the bacterial cell. They consist of a tetradecameric, proteolytic ClpP core and associated AAA+ Clp-ATPases. The Clp system of *Streptomyces* is unusually complex, comprising up to five ClpP homologs (ClpP1–ClpP5) and three Clp-ATPases (ClpX, ClpC1, and ClpC2). Streptomycetes produce a plethora of secondary metabolites, including potent acyldepsipeptide (ADEP) antibiotics, which target ClpP. We have previously reported on the operation mode of the *Streptomyces* ClpP1P2 protease and identified a novel *clpP* gene (named *clpP_ADEP_*) as a resistance determinant encoded near the ADEP biosynthesis gene cluster. However, the molecular function of ClpP_ADEP_ remains enigmatic. Here, we report on the molecular self-resistance mechanism to ADEP via ClpP_ADEP_ and its interaction with the Clp system in the ADEP producer *Streptomyces hawaiiensis* NRRL 15010. By combining cell-based and *in vitro* studies, we show that ClpP_ADEP_ interferes with the formation of the ClpP1P2 complex and inhibits the proteolytic activity of ClpP1. Moreover, ClpP_ADEP_ forms a functional complex with ClpP2 and Clp-ATPases. By these means, ClpP_ADEP_ protects the producer cell against ADEP in a two-pronged approach. On the one hand, it prevents ADEP from corrupting ClpP1 to degrade delicate essential proteins and polypeptides. On the other hand, ClpP_ADEP_ ensures the survival of the producer cells by maintaining the housekeeping function of the Clp protease in regulatory proteolysis.

**IMPORTANCE:**

Acyldepsipeptide (ADEP) antibiotics kill bacteria using an unusual mechanism of action, that is, the multilayered deregulation and activation of ClpP, the proteolytic core of the bacterial Clp protease. ADEP is highly effective in killing Gram-positive bacteria, including methicillin-resistant *Staphylococcus aureus* (MRSA) and vancomycin-resistant enterococci (VRE). Considering the elaborate mechanism of action of ADEP as well as the complexity of the essential Clp system in *Streptomyces* with up to five ClpP homologs as potential ADEP targets, the question arises: how does the producer ensure self-resistance in such a complex system? Here, we describe the molecular mechanism of self-resistance to ADEP in the producer *Streptomyces hawaiiensis* NRRL 15010, which is based on the presence of a phylogenetically distinct ClpP protein in the genome of the ADEP producer strain.

## OBSERVATION

Acyldepsipeptide (ADEP) antibiotics are highly active against many Gram-positive species, including multi-resistant staphylococci, streptococci, and enterococci, with minimum inhibitory concentrations in the sub-µg/mL range. Accordingly, ADEP showed high efficacy in treating staphylococcal, enterococcal, and streptococcal infections in rodents (1, 2), and in combination with rifampicin, even eradicated persister cells in chronic biofilm infections (3). ADEP targets and deregulates ClpP, the core of the bacterial caseinolytic protease Clp (4, 5). The Clp protease is ubiquitous in bacterial cells, where it plays a crucial role in regulatory proteolysis and protein homeostasis (6, 7). Clp is a self-compartmentalizing serine protease composed of the proteolytic, tetradecameric core ClpP and cognate, hexameric AAA+ unfoldases (i.e., Clp-ATPases, such as ClpX and ClpC in *Staphylococcus aureus*), which recognize, unfold, and feed substrates of the Clp system into the barrel-shaped ClpP tetradecamer (8, 9). In Firmicutes, ADEP inhibits regulatory proteolysis of natural Clp protein substrates by abrogating the interaction of the proteolytic core ClpP with its partner AAA+ Clp-ATPases (5, 10–12) while activating the ClpP core for uncontrolled proteolysis via direct binding and conformational control (13–16).

Bacteria exposed to ADEP under laboratory conditions develop resistance, which entails spontaneous mutations in ClpP affecting its oligomerization, conformation, catalytic function, or interaction with partnering Clp-ATPases or ADEP (3, 17–20). However, these mutations also impair the function of the Clp protease in regulatory proteolysis and protein homeostasis. Since loss of ClpP function severely attenuates the ability of multiple pathogenic bacteria to colonize and infect rodents, it is tempting to speculate that such spontaneous loss-of-function mutations will less frequently occur in the host environment (4, 17). Due to the complexity of the essential Clp system in *Streptomyces* (21) and the multi-layered activities of ADEP, spontaneous loss-of-function mutations can also not explain the self-resistance of the ADEP producer *Streptomyces hawaiiensis* NRRL 15010.

Streptomycetes possess, in fact, one of the most complex Clp machineries reported in eubacteria so far. For example, the genome of the model organism *Streptomyces lividans* (sl) encodes at least three Clp-ATPases (ClpX, ClpC1, and ClpC2) and five ClpP homologs (ClpP1–ClpP5) (21, 22), and individual ClpP proteins show high homology among different *Streptomyces* strains (Fig. S1A through E). In contrast, most other bacterial genomes encode a single or two ClpP homologs, the latter often interacting to form ClpP1P2 hetero-tetradecamers. We have recently provided molecular insights into the operation mode of the ClpP1P2 complex in *Streptomyces* (18). We have shown that a ClpP1P2 heterotetradecamer forms the proteolytic core of the housekeeping Clp protease, with ClpP1 mainly accountable for the proteolytic activity of the ClpP1P2 complex, and ClpP2 for the recruitment of the Clp-ATPases. One of the natural substrates of ClpP1P2 is the transcriptional activator of the *clpP3P4* operon, PopR. Its continuous degradation by ClpP1 (within the ClpP1P2 complex) ensures that the non-isofunctional ClpP3P4 core is expressed as a backup only in situations when ClpP1 or ClpP2 is not functioning (18, 23). Furthermore, while ClpP1 has been identified as a target for ADEP (18, 24), ClpP2 and ClpP3P4 were found to be ADEP-insensitive (18, 24), that is, ADEP binds to and deregulates ClpP1, but not ClpP2, and the interaction of ClpP2 with ClpX, ClpC1, or ClpC2 is not prevented by ADEP (18).

Recently, we identified a sixth *clpP* gene (named *clpP_ADEP_*) in the vicinity of the ADEP biosynthetic gene cluster in the ADEP producer *S. hawaiiensis* NRRL 15010 (25). Initial cell-based studies revealed that this accessory ClpP homolog acts as a resistance determinant against ADEP, conferring high-level ADEP resistance when heterologously expressed in ADEP-sensitive streptomycetes (25). However, the underlying molecular mechanism that leads to ADEP resistance remained unsolved. In this study, by combining cell-based and *in vitro* studies, we clarify the molecular basis of ClpP_ADEP_-mediated resistance, thereby revealing a novel mechanism of producer self-resistance.

### Degradation of ClpP1 or expression of ADEP-insensitive ClpP3 is not part of the self-resistance mechanism of the ADEP producer

To elucidate the mechanism of ClpP_ADEP_-mediated resistance, we first tested the hypothesis that ClpP_ADEP_ may interact with the ADEP target ClpP1 to inhibit its function. ClpP1 inactivation would allow *Streptomyces* to survive *via* accumulation of the ClpP1P2 substrate PopR, an activator of the expression of the ADEP-insensitive ClpP3P4 (21). Such a survival strategy was previously reported for spontaneous, ADEP-resistant *S. lividans* mutants, which carried loss-of-function mutations in the *clpP1* gene resulting in ClpP3P4 expression (24). Indeed, amino acid sequence alignments and phylogenetic analyses revealed that ClpP_ADEP_ shows the highest homology to ClpP1 (Fig. S1F), suggesting that an interaction between ClpP_ADEP_ and ClpP1 may be possible. Furthermore, we determined the transcript and protein levels of ClpP1, ClpP2, and ClpP_ADEP_ in the ADEP producer *S. hawaiiensis*. qPCR analysis revealed that *clpP_ADEP_* was expressed already in the early exponential phase and transcript levels of *clpP_ADEP_* and *clpP1* closely paralleled each other throughout the different growth phases of the producer culture, while *clpP2* transcript levels were slightly lower (Fig. S2). The protein level of ClpP_ADEP_, detected by quantitative proteomics, was considerable and reached approximately half the amount recorded for ClpP1 and ClpP2 (Fig. S3).

**Fig 1:**
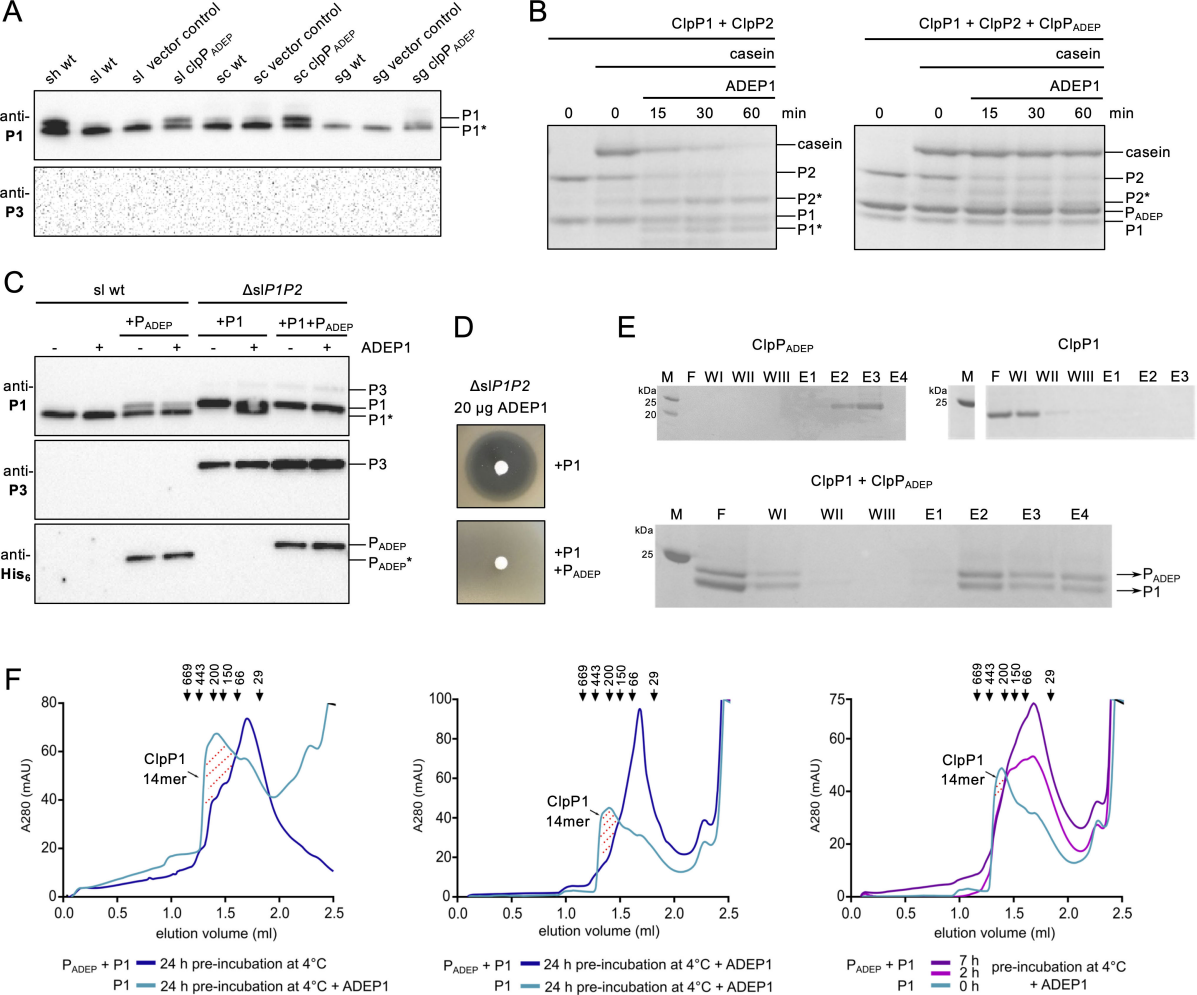
ClpP_ADEP_ disrupts toxic, ADEP-activated ClpP1 complexes. (**A**). Immunoblotting of cell extracts of *S. hawaiiensis* wild type (sh wt) as well as wild type or ClpP_ADEP_-expressing mutants of *S. lividans* (sl), *S. coelicolor* (sc), and *S. griseus* (sg). Anti-*Streptomyces* ClpP1 (anti-P1) or ClpP3 (anti-P3) antibodies were used as indicated. ClpP3 expression was used as a readout for ClpP1 inactivation. Vector control, pSETermE* (**B**). *In vitro* protease activity assays using heterologously expressed and isolated *S. hawaiiensis* ClpP proteins and casein as a model substrate. See Fig. S5 for replicate experiments. (**C**). Immunoblotting of cell extracts of ADEP-treated *S. lividans* wild type (sl wt) and *S. lividans* Δ*P1P2* (Δsl*P1P2*) complemented with sl*clpP1* and/or *clpP_ADEP_* as indicated. Strains were grown for 24 h and subsequently treated (+) with 5 µg/mL of ADEP1 for another 3 hours, upon which crude cell extracts were prepared and analyzed by immunoblotting using anti-P1, anti-P3, or anti-His_6_ antibodies. ClpP3 expression was used as a readout for ClpP1 inactivation. (**D**). ADEP disk diffusion bioassay of *S. lividans* Δ*P1P2* complemented with either sl*clpP1* or both sl*clpP1* and *clpP_ADEP_*. (**E**). Pull-down experiment applying pre-incubated mixtures of untagged shClpP1 and ClpP_ADEP_-His_6_ to nickel-nitrilotriacetic acid agarose. SDS-PAGE of flow-through (**F**), wash steps (W I–III), and elution fractions (E 1–4), M, protein marker. See Fig. S6 for full-size SDS PAGE. (**F**). Size exclusion chromatography of purified *S. hawaiiensis* ClpP1 and ClpP_ADEP_ proteins in the absence or presence of ADEP1. For a size exclusion experiment demonstrating the conformational control that ADEP exerts on ClpP1, that is, inducing the active, extended conformation of ClpP1, see Fig. 3H in Reinhardt et al. (18). P1, unprocessed ClpP1; P1*, processed ClpP1; P2, unprocessed ClpP2; P2*, processed ClpP2; P_ADEP_, ClpP_ADEP_.

To investigate whether ClpP1 inactivation might occur by degradation, we analyzed cell extracts of the ADEP-producer *S. hawaiiensis* (sh), as well as of wild-type *S. lividans* (sl), *Streptomyces coelicolor* (sc), and *Streptomyces griseus* (sg), and ADEP-resistant mutants thereof that heterologously expressed ClpP_ADEP_. Western Blot analysis revealed the full-length ClpP1 protein and its naturally processed form ClpP1* in the extracts of all the strains (Fig. 1A). Thus, ClpP_ADEP_-mediated detoxification of ClpP1 does not occur by proteolytic degradation. Of note, *Streptomyces* ClpP proteins undergo processing reactions both *in vitro* and in living cells (18). As shown previously, ClpP1 processing requires the presence of ClpP2 and a Clp activator (e.g., either a Clp-ATPase interacting with ClpP2 or ADEP interacting with ClpP1), whereas ClpP2 processing depends exclusively on the presence of ClpP1, but not on a Clp-ATPase or ADEP. However, the processing of both ClpP1 and ClpP2 depends entirely on the integrity of the ClpP1 catalytic triad (18). In our current study, we noted that unprocessed ClpP1 occurred in higher amounts in extracts of cells that expressed ClpP_ADEP_, suggesting a ClpP_ADEP_-mediated inhibition of ClpP1 self-processing. For more details on the processing of ClpP proteins in *Streptomyces*, the reader is kindly referred to our recent publication by Reinhardt et al. (18).

Notably, in the same Western blot assay, we also used an anti-*Streptomyces* ClpP3 (anti-P3) antibody as a readout for ClpP1 inactivation and did not detect ClpP3 expression, which indicates that the PopR-degrading natural function of the ClpP1P2 complex was maintained in the ADEP producer. Likewise, ClpP3 was absent in ADEP-resistant *S. lividans*, *S. coelicolor*, and *S. griseus* that heterologously expressed ClpP_ADEP_ (Fig. 1A).

### ClpP_ADEP_ confers ADEP resistance by disrupting ADEP-activated ClpP1 complexes

Next, we conducted an *in vitro* protease activity assay using heterologously expressed ClpP proteins and casein as a model substrate (Fig. 1B). While casein was efficiently degraded by ClpP1P2 in the presence of ADEP, the addition of ClpP_ADEP_ inhibited processing of ClpP1 as well as casein degradation by ADEP-activated ClpP1P2, indicating a direct effect of ClpP_ADEP_ on the function of ClpP1.

To further examine a potential effect of ClpP_ADEP_ on the function of ClpP1 in *Streptomyces* cells, we analyzed the effects of inducible expression of *clpP_ADEP_* in *S. lividans* wild type and of *slclpP1 and clpP_ADEP_* in a Δ*P1P2* background, that is, in the presence or absence of ClpP2, respectively (Fig. 1C). Here, using an anti-P1 antibody, we confirmed that the expression of ClpP_ADEP_ slows down auto-processing of ClpP1 in *S. lividans* wild type, since bands of unprocessed ClpP1 could still be detected. In the *S. lividans* Δ*P1P2* background, ClpP_ADEP_ entirely prevented ADEP-induced ClpP1 auto-processing, and ClpP_ADEP_ itself was only detected in its unprocessed form.

Notably, in *S. lividans* Δ*P1P2*, we detected ClpP3 when either ClpP1 or ClpP_ADEP_ alone or both were introduced, but ClpP2 was lacking. These results indicate that although ClpP_ADEP_ must interact with ClpP1 to affect its functions, no proteolytically active complex is formed between these two ClpP proteins that could substitute for the housekeeping functions of the ClpP1P2 complex. Visible proof is the induction of ClpP3 expression, as a readout for ClpP1 inactivation, and the failed processing of ClpP1 and ClpP_ADEP_.

Of note, using an ADEP bioassay, *S. lividans* Δ*P1P2* was found to be sensitive to ADEP treatment when complemented with ClpP1 alone, showing that the antibacterial activity of ADEP does not require ClpP2, while *S. lividans* Δ*P1P2* was ADEP-resistant when complemented with both ClpP1 and ClpP_ADEP_ (Fig. 1D).

To obtain more information about the nature of the interaction between ClpP1 and ClpP_ADEP_, we pre-incubated a mixture of untagged shClpP1 with ClpP_ADEP_-His_6_ followed by pull-down with nickel-nitrilotriacetic acid. Here, despite extensive washing with 20 mM imidazole, we observed co-elution of both shClpP1 and ClpP_ADEP_-His_6_ in a similar ratio, indicating a direct interaction of both homologs (Fig. 1E). In addition, we examined complex formation between shClpP1 and ClpP_ADEP_ in the absence or presence of ADEP by size exclusion chromatography (Fig. 1F). Pre-incubation of shClpP1 with ADEP induced the formation of tetradecameric shClpP1 complexes as reported previously (18). However, the presence of ClpP_ADEP_ prevented ADEP-induced tetradecamer formation of shClpP1, and only ClpP oligomers smaller than 70 kDa could form, representing ClpP dimers and/or trimers. Of note, ClpP_ADEP_ alone eluted mostly in lower oligomeric states (Fig. S4). Hence, the interplay of ClpP1 and ClpP_ADEP_ involves a strong, direct interaction of both homologs resulting in mixed ClpP1/ClpP_ADEP_ dimers and trimers, thereby preventing the formation of catalytically active, tetradecameric ClpP complexes of both homomeric and heteromeric nature, even in the presence of the tetradecamer-inducing activator ADEP.

### ClpP_ADEP_ ensures natural substrate degradation by forming an ADEP-insensitive ClpP_ADEP_P2-Clp-ATPase complex

**Fig 2:**
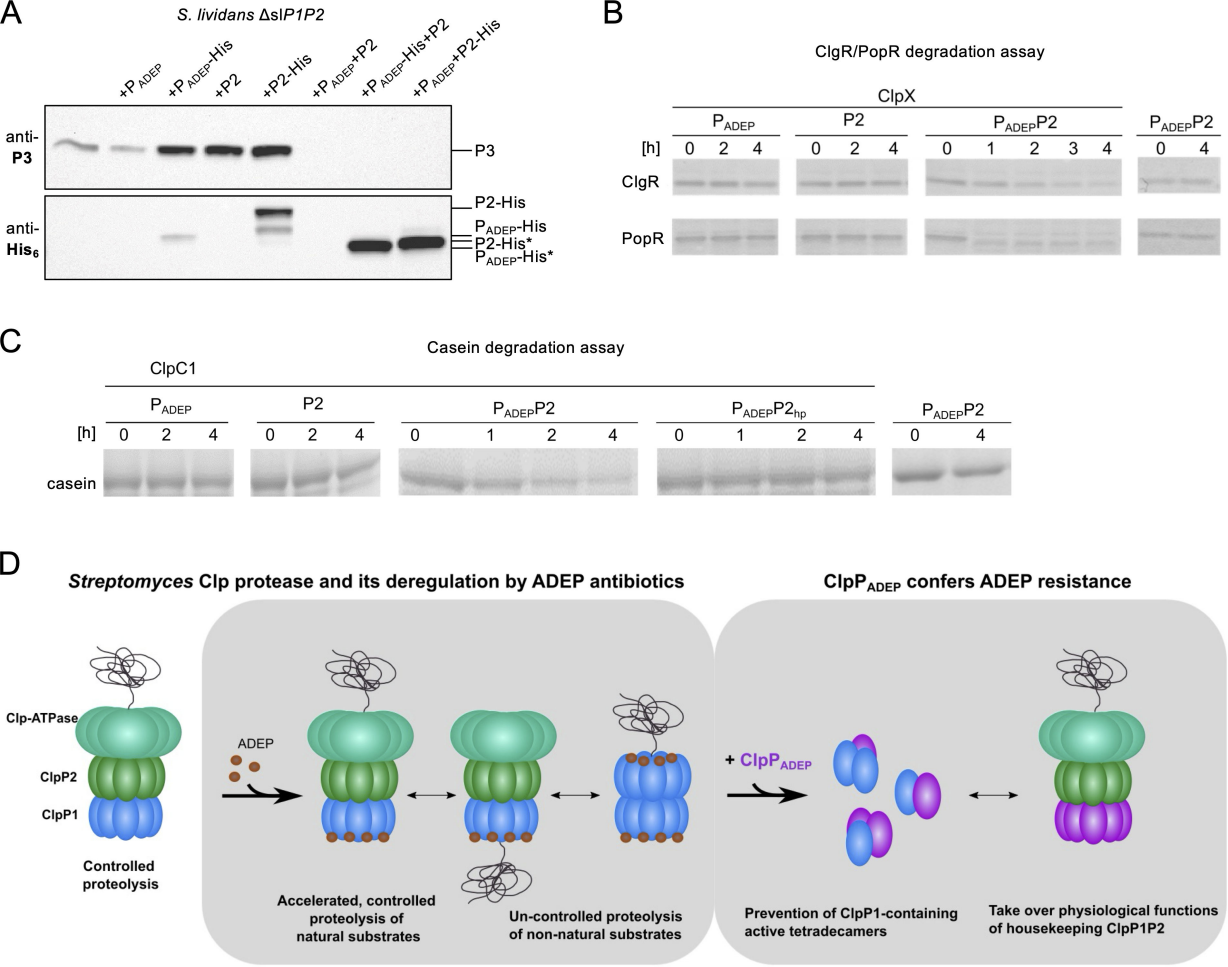
ClpP_ADEP_ ensures natural substrate degradation by the formation of an ADEP-insensitive ClpP_ADEP_P2-Clp-ATPase complex. (**A**) Immunoblotting of cell extracts of *S. lividans* Δ*P1P2* complemented with either *clpP_ADEP_*, *slclpP2*, or both, as indicated. Strains were grown for 24 h and crude cell extracts were prepared and analyzed by immunoblotting using anti-P3 and anti-His_6_ antibodies. ClpP3 expression was used as a readout for ClpP1 inactivation. Of note, in *S. lividans* Δ*P1P2*, a double band is detected for ClpP2-His using anti-His_6_ antibodies as previously observed (18). (**B**) *In vitro* protease activity assays using heterologously expressed and isolated *S. hawaiiensis* proteins ClpP_ADEP_ and ClpP2, the Clp-ATPase protein ClpX, as well as the natural ClpXP1P2 substrates ClgR and PopR. See Fig. S7 for replicate experiments. (**C**) *In vitro* protease activity assays using the isolated *S. hawaiiensis* proteins ClpP_ADEP_ and ClpP2, the Clp-ATPase protein ClpC1, as well as the model protein substrate β-casein. In addition, a ClpP2 hydrophobic pocket mutant (ClpP2_hp_) was used to analyze Clp-ATPase binding. See Fig. S8 for replicate experiments. (**D**) Model of ADEP mode of action and mode of resistance in *Streptomyces*. For further details on the mode of action, see (18). ClpP_ADEP_ prevents the formation of toxic ClpP1-containing tetradecamers and forms a new proteolytic core with ClpP2 to take over essential natural functions. P2, unprocessed ClpP2; P2*, processed ClpP2; P_ADEP_, unprocessed ClpP_ADEP_; P_ADEP_*, processed ClpP_ADEP_.

Although our data indicated that ClpP_ADEP_ interferes with ClpP1 oligomerization and activity (Fig. 1B, E, and F), important cellular functions, which commonly rely on an operational ClpP1P2 complex, appeared to run unhindered in *S. hawaiiensis*. A clear indication was the lack of ClpP3 expression in ClpP_ADEP_-containing cells (Fig. 1A and C). Therefore, we next explored a putative interaction of ClpP_ADEP_ with ClpP2 in *Streptomyces* cells in the absence of slClpP1 by complementing *S. lividans* Δ*P1P2* with *clpP_ADEP_*, *slclpP2*, or both. To allow detection of ClpP_ADEP_ or ClpP2 in immunoblots, proteins were expressed as either untagged or His_6_-tagged versions. Then, we used these strains in immunoblotting experiments to study ClpP3 expression (Fig. 2A). Once again, the absence of ClpP3 served as a proxy for a functional ClpP complex capable of degrading PopR, the transcriptional activator of the *clpP3P4* operon (18). Interestingly, ClpP3 expression was observed when either ClpP_ADEP_ or ClpP2 was present alone, but ClpP3 expression was lost when both ClpP_ADEP_ and ClpP2 were co-expressed. Furthermore, ClpP homologs were only processed when ClpP_ADEP_ and ClpP2 were co-expressed, as indicated by anti-His_6_ antibody detection. These cell-based data implied that ClpP_ADEP_ and ClpP2 form a proteolytically active, heteromeric complex, which allows processing of both ClpP_ADEP_ and ClpP2 and finally enables the degradation of natural ClpP1P2 substrates, for example, PopR, as indicated by the repressed expression of ClpP3.

To corroborate the results of our cell-based experiments, we performed *in vitro* protease activity assays using the isolated *S. hawaiiensis* proteins ClpP_ADEP_ and ClpP2, the cognate Clp-ATPase ClpX, as well as the natural ClpXP1P2 substrates ClgR and PopR. When testing different combinations of ClpP_ADEP_, shClpP2, and ClpX to attempt the digestion of ClgR and PopR (Fig. 2B), we found that neither ClpP2 nor ClpP_ADEP_ alone was capable of protein degradation with ClpX. However, when ClpP_ADEP_, ClpP2, and ClpX were combined, ClgR and PopR were digested, thus validating that ClpP_ADEP_ and ClpP2 jointly substitute for the ClpP1P2 core in natural protein degradation. Likewise, when we used the Clp-ATPase ClpC1 in *in vitro* degradation assays with the model protein substrate ß-casein, proteolysis occurred only when ClpP_ADEP_, ClpP2, and ClpC1 were combined (Fig. 2C). Of note, when ClpP2 was mutated in its Clp-ATPase interaction site, the hydrophobic pocket, and thereby rendered incapable of Clp-ATPase binding (18), the degradation of ß-casein was prevented. Our data, therefore, consistently show that ClpP_ADEP_ and ClpP2 form a proteolytically active heteromeric Clp protease core that interacts with either ClpX or ClpC1, with ClpP2 representing the interaction partner for the Clp-ATPases.

In summary, our data unravel the molecular basis of a novel mechanism of producer self-resistance. Here, the mode of action of the ClpP-deregulating ADEP antibiotic is matched by a self-resistance mechanism conferred by ClpP_ADEP_, which is far more complex than simple target mutation (Fig. 2D). The presence of an extra, ADEP-resistant ClpP_ADEP_ protein protects the cell from the detrimental consequences of ADEP by (i) preventing tetradecamer formation of the ADEP target ClpP1 and consequently inhibiting its catalytic activity and (ii) substituting for ClpP1 in a complex with ClpP2 and a Clp-ATPase to preserve the housekeeping functions of the Clp protease in regulatory proteolysis and protein homeostasis, even in the presence of ADEP antibiotics. The combination of these diverse abilities in a single protein is remarkable, as it requires direct interaction with two different ClpP partners, on the one hand, preventing and, on the other hand, newly generating catalytic function.

One might wonder why this rather complex resistance mechanism evolved in the ADEP producer instead of a mere out-of-function point mutation in the ADEP target ClpP1. A plausible explanation could be a diverging substrate spectrum of the non-isofunctional pairs ClpP1P2 and ClpP3P4, probably mediated or enhanced by a distinct interaction preference for the Clp-ATPases. The full substrate spectrum of those *Streptomyces* proteases is elusive, but it was shown that an *S. lividans clpP1* mutant had a cell cycle defect (21). It presented a “bald” phenotype by arrest in the basal mycelial state and loss of sporulation capacity. Expressing *clpP3* and *clpP4* in this strain did not rescue the bald phenotype (21). *Vice versa*, a *clpP3* mutant was not affected in its cell cycle but produced more of the blue antibacterial pigment actinorhodin (21).

Introducing the phylogenetically distinct ClpP_ADEP_ to modulate the multi-layered Clp system in *Streptomyces* seems to have been overall worthwhile, but still a challenge for nature, as suggested by the fact that *S. hawaiiensis* NRRL 15010 is the only known ADEP producer known to date.

## SUPPLEMENTAL MATERIAL

Fig. S1 mbio.01652-25-s0001.pdf Multiple amino acid sequence alignment and phylogenetic tree analyses of ClpP proteins from *Streptomyces* species.

Fig. S2 mbio.01652-25-s0002.pdf Relative transcript levels of clpP1-P5 and clpPADEP in *S. hawaiiensis*.

Fig. S3 mbio.01652-25-s0003.pdf Quantitative proteomic analysis of ClpP1, ClpP2 and ClpPADEP in *S. hawaiiensis* and *S. lividans*.

Fig. S4 mbio.01652-25-s0004.pdf Size exclusion chromatography of ShClpPADEP.

Fig. S5 mbio.01652-25-s0005.pdf Casein degradation assays.

Fig. S6 mbio.01652-25-s0006.pdf Pull-down experiment of shClpP1 and ClpPADEP-His6.

Fig. S7 mbio.01652-25-s0007.pdf Protein degradation assays: ClgR/PopR.

Fig. S8 mbio.01652-25-s0008.pdf Protein degradation assays: casein.

Supplemental text mbio.01652-25-s0009.pdf Supplemental methods.

Table S1 mbio.01652-25-s0010.pdf Bacterial strains and plasmids.

ASM does not own the copyrights to Supplemental Material that may be linked to, or accessed through, an article. The authors have granted ASM a non-exclusive, world-wide license to publish the Supplemental Material files. Please contact the corresponding author directly for reuse.

## References

[1] Brötz-Oesterhelt H, Beyer D, Kroll H-P, Endermann R, Ladel C, Schroeder W, Hinzen B, Raddatz S, Paulsen H, Henninger K, Bandow JE, Sahl H-G, Labischinski H. 2005. Dysregulation of bacterial proteolytic machinery by a new class of antibiotics. Nat Med 11:1082–1087. doi:10.1038/nm1306 16200071

[2] Brown Gandt A, Griffith EC, Lister IM, Billings LL, Han A, Tangallapally R, Zhao Y, Singh AP, Lee RE, LaFleur MD. 2018. In vivo and in vitro effects of a ClpP-activating antibiotic against vancomycin-resistant enterococci. Antimicrob Agents Chemother 62:e00424-18. doi:10.1128/AAC.00424-18 29784838 PMC6105829

[3] Conlon BP, Nakayasu ES, Fleck LE, LaFleur MD, Isabella VM, Coleman K, Leonard SN, Smith RD, Adkins JN, Lewis K. 2013. Activated ClpP kills persisters and eradicates a chronic biofilm infection. Nature 503:365–370. doi:10.1038/nature12790 24226776 PMC4031760

[4] Brötz-Oesterhelt H, Vorbach A. 2021. Reprogramming of the caseinolytic protease by ADEP antibiotics: molecular mechanism, cellular consequences, therapeutic potential. Front Mol Biosci 8:690902. doi:10.3389/fmolb.2021.690902 34109219 PMC8182300

[5] Brötz-Oesterhelt H, Sass P. 2014. Bacterial caseinolytic proteases as novel targets for antibacterial treatment. Int J Med Microbiol 304:23–30. doi:10.1016/j.ijmm.2013.09.001 24119566

[6] Olivares AO, Baker TA, Sauer RT. 2016. Mechanistic insights into bacterial AAA+ proteases and protein-remodelling machines. Nat Rev Microbiol 14:33–44. doi:10.1038/nrmicro.2015.4 26639779 PMC5458636

[7] Illigmann A, Thoma Y, Pan S, Reinhardt L, Brötz-Oesterhelt H. 2021. Contribution of the Clp protease to bacterial survival and mitochondrial homoeostasis. Microb Physiol 31:260–279. doi:10.1159/000517718 34438398

[8] Gottesman S, Roche E, Zhou Y, Sauer RT. 1998. The ClpXP and ClpAP proteases degrade proteins with carboxy-terminal peptide tails added by the SsrA-tagging system. Genes Dev 12:1338–1347. doi:10.1101/gad.12.9.1338 9573050 PMC316764

[9] Baker TA, Sauer RT. 2012. ClpXP, an ATP-powered unfolding and protein-degradation machine. Biochim Biophys Acta 1823:15–28. doi:10.1016/j.bbamcr.2011.06.007 21736903 PMC3209554

[10] Malik IT, Brötz-Oesterhelt H. 2017. Conformational control of the bacterial Clp protease by natural product antibiotics. Nat Prod Rep 34:815–831. doi:10.1039/c6np00125d 28375422

[11] Famulla K, Sass P, Malik I, Akopian T, Kandror O, Alber M, Hinzen B, Ruebsamen-Schaeff H, Kalscheuer R, Goldberg AL, Brötz-Oesterhelt H. 2016. Acyldepsipeptide antibiotics kill mycobacteria by preventing the physiological functions of the ClpP1P2 protease. Mol Microbiol 101:194–209. doi:10.1111/mmi.13362 26919556 PMC5469208

[12] Kirstein J, Hoffmann A, Lilie H, Schmidt R, Rübsamen-Waigmann H, Brötz-Oesterhelt H, Mogk A, Turgay K. 2009. The antibiotic ADEP reprogrammes ClpP, switching it from a regulated to an uncontrolled protease. EMBO Mol Med 1:37–49. doi:10.1002/emmm.200900002 20049702 PMC3378108

[13] Gersch M, Famulla K, Dahmen M, Göbl C, Malik I, Richter K, Korotkov VS, Sass P, Rübsamen-Schaeff H, Madl T, Brötz-Oesterhelt H, Sieber SA. 2015. AAA+ chaperones and acyldepsipeptides activate the ClpP protease via conformational control. Nat Commun 6:6320. doi:10.1038/ncomms7320 25695750

[14] Lee B-G, Park EY, Lee K-E, Jeon H, Sung KH, Paulsen H, Rübsamen-Schaeff H, Brötz-Oesterhelt H, Song HK. 2010. Structures of ClpP in complex with acyldepsipeptide antibiotics reveal its activation mechanism. Nat Struct Mol Biol 17:471–478. doi:10.1038/nsmb.1787 20305655

[15] Sass P, Josten M, Famulla K, Schiffer G, Sahl HG, Hamoen L, Brötz-Oesterhelt H. 2011. Antibiotic acyldepsipeptides activate ClpP peptidase to degrade the cell division protein FtsZ. Proc Natl Acad Sci USA 108:17474–17479. doi:10.1073/pnas.1110385108 21969594 PMC3198362

[16] Silber N, Pan S, Schäkermann S, Mayer C, Brötz-Oesterhelt H, Sass P. 2020. Cell division protein FtsZ is unfolded for N-terminal degradation by antibiotic-activated ClpP. mBio 11:e01006-20. doi:10.1128/mBio.01006-20 32605984 PMC7327170

[17] Malik IT, Pereira R, Vielberg MT, Mayer C, Straetener J, Thomy D, Famulla K, Castro H, Sass P, Groll M, Brötz-Oesterhelt H. 2020. Functional characterisation of ClpP mutations conferring resistance to acyldepsipeptide antibiotics in firmicutes. Chembiochem 21:1997–2012. doi:10.1002/cbic.201900787 32181548 PMC7496096

[18] Reinhardt L, Thomy D, Lakemeyer M, Westermann LM, Ortega J, Sieber SA, Sass P, Brötz-Oesterhelt H. 2022. Antibiotic acyldepsipeptides stimulate the Streptomyces Clp-ATPase/ClpP complex for accelerated proteolysis. mBio 13:e01413-22. doi:10.1128/mbio.01413-22 36286522 PMC9765437

[19] Pan S, Malik IT, Thomy D, Henrichfreise B, Sass P. 2019. The functional ClpXP protease of Chlamydia trachomatis requires distinct clpP genes from separate genetic loci. Sci Rep 9:14129. doi:10.1038/s41598-019-50505-5 31575885 PMC6773864

[20] Pan S, Jensen AA, Wood NA, Henrichfreise B, Brötz-Oesterhelt H, Fisher DJ, Sass P, Ouellette SP. 2023. Molecular characterization of the ClpC AAA+ ATPase in the biology of Chlamydia trachomatis. mBio 14:e00075-23. doi:10.1128/mbio.00075-23 36975997 PMC10128030

[21] Viala J, Rapoport G, Mazodier P. 2000. The clpP multigenic family in Streptomyces lividans: conditional expression of the clpP3 clpP4 operon is controlled by PopR, a novel transcriptional activator. Mol Microbiol 38:602–612. doi:10.1046/j.1365-2958.2000.02155.x 11069683

[22] de Crécy-Lagard V, Servant-Moisson P, Viala J, Grandvalet C, Mazodier P. 1999. Alteration of the synthesis of the Clp ATP-dependent protease affects morphological and physiological differentiation in Streptomyces. Mol Microbiol 32:505–517. doi:10.1046/j.1365-2958.1999.01364.x 10320574

[23] Viala J, Mazodier P. 2002. ClpP-dependent degradation of PopR allows tightly regulated expression of the clpP3 clpP4 operon in Streptomyces lividans. Mol Microbiol 44:633–643. doi:10.1046/j.1365-2958.2002.02907.x 11994147

[24] Gominet M, Seghezzi N, Mazodier P. 2011. Acyl depsipeptide (ADEP) resistance in Streptomyces. Microbiology (Reading) 157:2226–2234. doi:10.1099/mic.0.048454-0 21636652

[25] Thomy D, Culp E, Adamek M, Cheng EY, Ziemert N, Wright GD, Sass P, Brötz-Oesterhelt H. 2019. The ADEP biosynthetic gene cluster in Streptomyces hawaiiensis NRRL 15010 reveals an accessory clpP gene as a novel antibiotic resistance factor. Appl Environ Microbiol 85:e01292-19. doi:10.1128/AEM.01292-19 31399403 PMC6805094

