# Supplementary material for: Self-resistance mechanism to acyldepsipeptide antibiotics in the Streptomyces producer
Source: mBio. 2025 Oct 6;16(11):e01652-25. doi: 10.1128/mbio.01652-25 (PMC12607617; doi:10.1128/mbio.01652-25)

SI file

Self-resistance mechanism to acyldepsipeptide antibiotics in the *Streptomyces* producer

Dhana Thomy[1,2,4], Laura Reinhardt[1,2,4], Elisa Liebhart[1,2], Mirita Franz-Wachtel[2,3], Boris Maček[2,3], Peter Sass[1,2+], Heike Brötz-Oesterhelt[1,2*+].
[1]Department of Microbial Bioactive Compounds, IMIT, University of Tübingen, Germany. [2]Cluster of Excellence - Controlling Microbes to Fight Infections, University of Tübingen, Germany. [3]Proteome Center Tübingen, University of Tübingen, Germany. [4]Dhana Thomy and Laura Reinhardt contributed equally to this work. Author order was determined by seniority. *heike.broetz-oesterhelt@uni-tuebingen.de.
[+]Peter Sass and Heike Brötz-Oesterhelt share senior authorship.

A

ClpP1 Multiple sequence alignment (CLUSTAL O, v1.2.4, EMBL-EBI)

```
SgClpP1         --------------MTNLMPYAAGEPSLGGGLGDQVYSRLLGERIIFLGQQVDDDIANKI   46
ShClpP1         MRRPGAVVRRAGGYVTNLMPSAAGEPSIGGGLGDQVYNRLLNERIIFLGQPVDDDIANKI   60
SlClpP1         MRRPGAVVRRAGGYVTNLMPSAAGEPSIGGGLGDQVYNRLLGERIIFLGQPVDDDIANKI   60
ScClpP1         MRRPGAVVRRAGGYVTNLMPSAAGEPSIGGGLGDQVYNRLLGERIIFLGQPVDDDIANKI   60
                              :***** ******:*********.***.******** *********

SgClpP1         TAQLLLLAAEPDKDIYLYINSPGGSVTAGMAVYDTMQYIPNDVVTIGMGMAASMGQFLLT   106
ShClpP1         TAQLLLLASDPEKDIYLYINSPGGSITAGMAIYDTMQYIKNDVVTIAMGLAASMGQFLLS   120
SlClpP1         TAQLLLLASDPDKDIFLYINSPGGSITAGMAIYDTMQYIKNDVVTIAMGLAASMGQFLLS   120
ScClpP1         TAQLLLLASDPDKDIFLYINSPGGSITAGMAIYDTMQYIKNDVVTIAMGLAASMGQFLLS   120
                ********::*:***:*********:*****:******* ******.**:*********:

SgClpP1         GGAAGKRFALPNTDILMHQGSAGIGGTASDIKIQAQYLLRTKTRMAEITAHHSGQTVETI   166
ShClpP1         AGTPGKRFALPNAEILIHQPSAGLAGSASDIKIHAERLLHTKKRMAELTSQHTGQTIEQI   180
SlClpP1         AGTPGKRFALPNAEILIHQPSAGLAGSASDIKIHAERLLHTKRRMAELTSQHTGQTIEQI   180
ScClpP1         AGTPGKRFALPNAEILIHQPSAGLAGSASDIKIHAERLLHTKRRMAELTSQHTGQTIEQI   180
                .*: ********::**:** ***:.*:******:*: **:** ****:*::*:***:* *

SgClpP1         IRDGDRDRWYTAEEAKDYGLIDEIITVASGIPGGGGTGA        205
ShClpP1         TRDSDRDRWFDAFEAKEYGLIDDVMTTAAGMPGGGGTGA        219
SlClpP1         TRDSDRDRWFDAFEAKEYGLIDDVIATAAGMPGGGGTGA        219
ScClpP1         TRDSDRDRWFDAFEAKEYGLIDDVIATAAGMPGGGGTGA        219
                 **.*****: * ***:*****::::.*:*:********
```

Phylogenetic tree (CLUSTAL O, v1.2.4)

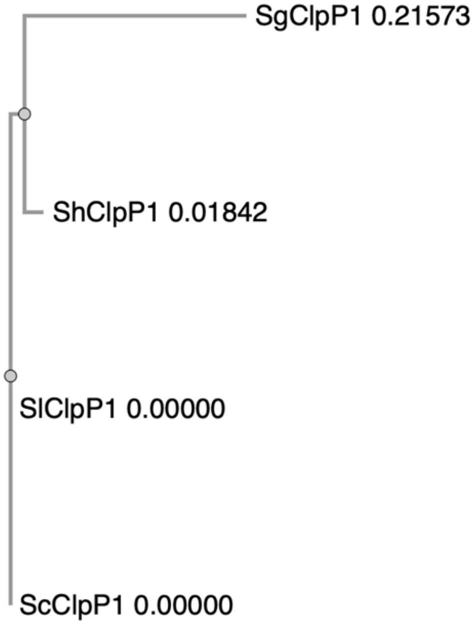

Figure S1. Multiple amino acid sequence alignment and phylogenetic tree analyses of ClpP proteins from *Streptomyces* spec (A-F). Alignments and phylogenetic analyses reveal high homology of individual ClpP proteins and show that $ClpP_{ADEP}$ has the highest homology to ClpP1, suggesting that an interaction between $ClpP_{ADEP}$ and ClpP1 may be possible. Data was analyzed using CLUSTAL O, v1.2.4, EMBL-EBI (Madeira F, Madhusoodanan N, Lee J, et al. The EMBL-EBI Job Dispatcher sequence analysis tools framework in 2024. Nucleic Acids Research. 52(W1):W521-W525, 2024). *Streptomyces hawaiiensis* ATCC 12236 (GenBank: CP021978.1), *Streptomyces lividans* TK24 (GenBank: CP009124.1), *Streptomyces coelicolor* A3(2 (Genbank: NC_003888.3), *Streptomyces griseus subsp. griseus* ATCC 12648 (GenBank: LVHX01000220.1). Sequences appear in aligned order. *, identical amino acid, :, conserved amino acid, ., semi-conserved amino acid. Predicted GUG initiating codons (instead of AUG) are underlined.

B

ClpP2 Multiple sequence alignment (CLUSTAL O, v1.2.4, EMBL-EBI)

```
SgClpP2      ---------MVNTHMNNFSGASASGLYTGPQVDNRYVVPRFVERTSQGVREYDPYAKLFE      51
ShClpP2      MNQFPGSGIYDRMHAVQDMS-ASQGRYTGPQAESRYIIPRFVERTSQGIREYDPYAKLFE      59
SlClpP2      MNDFPGSGLYDRVNAAQDMRAASQGRYTGPQAESRYVIPRFVERTSQGVREYDPYAKLFE      42
ScClpP2      MNDFPGSGLYDRVNAAQDMRAASQGRYTGPQAESRYVIPRFVERTSQGVREYDPYAKLFE      60
                        . :  :    ::.* *****.:.**::**********:***********

SgClpP2      ERVIFLGVQIDDASANDVMAQLLCLESMDPDRDISIYINSPGGSFTALTAIYDTMQFVKP      111
ShClpP2      ERVIFLGVQIDDASANDVMAQLLCLESMDPDRDISVYINSPGGSFTALTAIYDTMQYVKP      119
SlClpP2      ERVIFLGVQIDDASANDVMAQLLCLESMDPDRDISVYINSPGGSFTALTAIYDTMQYVKP      102
ScClpP2      ERVIFLGVQIDDASANDVMAQLLCLESMDPDRDISVYINSPGGSFTALTAIYDTMQYVKP      120
             ***********************************:********************:***

SgClpP2      DIQTVCMGQAASAAAVLLAAGTPGKRMALPHARVLIHQPSSQTGREQLSDLEIAANEILR      171
ShClpP2      DVQTVCMGQAASAAAVLLAAGTPGKRMALPNARVLIHQPYSETGRGQVSDLEIAANEILR      179
SlClpP2      DVQTVCMGQAASAAAVLLAAGTPGKRMALPNARVLIHQPYSETGRGQVSDLEIAANEILR      162
ScClpP2      DVQTVCMGQAASAAAVLLAAGTPGKRMALPNARVLIHQPYSETGRGQVSDLEIAANEILR      180
             *:****************************:******** *:*** *:************

SgClpP2      MRSQLEEMLARHSTTPLEKIREDIERDKILTAEDALAYGLVDQIVSTRKTTAGASL   227
ShClpP2      MRSQLEDMLAKHSTTPVEKIREDIERDKILTAEDALSYGLIDQVISTRKMDNSSLR   235
SlClpP2      MRSQLEEMLAKHSTTPVEKIREDIERDKILTAEDALSYGLIDQIITTRKMDNSSLR   218
ScClpP2      MRSQLEEMLAKHSTTPVEKIREDIERDKILTAEDALSYGLIDQIITTRKMDNSSLR   236
             ******:***:*****:*******************:***:**:::***   .:
```

Phylogenetic tree (CLUSTAL O, v1.2.4)

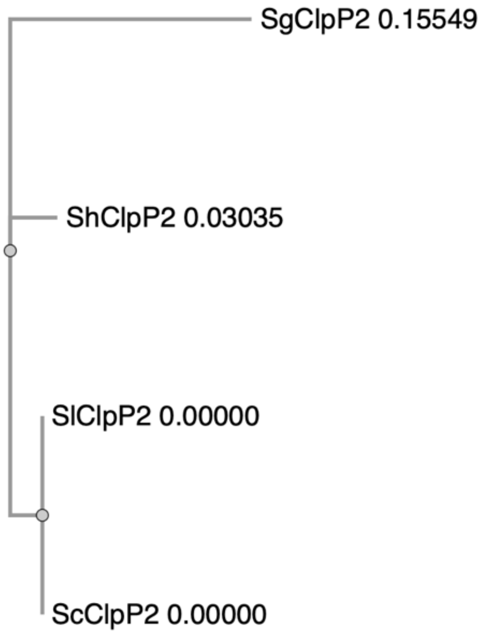

C

ClpP3 Multiple sequence alignment (CLUSTAL O, v1.2.4, EMBL-EBI)

```
ShClpP3      -----MTPLTALAPRAEEGDTPPTRFDDQLAAQLLAQRIVLLGTQVDEVSANRVCSQLLI      55
SlClpP3      MSPFTAGPAPARTPRAEEGDTPATRFDDRLAEQLLDQRIVLLGTQVDEVSANRVCAQLLI      60
ScClpP3      MSPFTAGPAPARTPRAEEGDTPATRFDDRLAEQLLDQRIVLLGTQVDEVSANRVCAQLLI      60
                    *  * :********* *****:** *** *******************:****

ShClpP3      LSAEDAHTDISLYINSPGGAVHAGLAIYDTMRLIPNDVSTLAMGFAASMGQFLLSVGAPG      115
SlClpP3      LSAQDPRTDISLYVNSPGGSVHAGLAIYDTMRLIPNDVSTLAMGFAASMGQFLLSVGTAG      120
ScClpP3      LSAQDPRTDISLYVNSPGGSVHAGLAIYDTMRLIPNDVSTLAMGFAASMGQFLLSVGTAG      120
             ***:* :******:*****:*************************************: *

ShClpP3      KRYALPNARIMMHQPSAGIGGTTADIEIQAQNLEFTKRTIERITAEHTGQSPENISRDGD      175
SlClpP3      KRYALPNARIMMHQPSAGIGGTTADIEIQADNLDFTKRTIERITAEHTGQSPETISRDGD      180
ScClpP3      KRYALPNARIMMHQPSAGIGGTTADIEIQADNLDFTKRTIERITAEHTGQSPETISRDGD      180
             ******************************:**:*******************.******

ShClpP3      RDRWFTAEEAREYGMVDRVVESLSDVRPAATRRRMGLQ  213
SlClpP3      RDRWFTAEEAREYGMVDQVVQSLADVRPAATRRRMGL-  217
ScClpP3      RDRWFTAEEAREYGMVDQVVQSLADVRPAATRRRMGL-  217
             *****************:**:**:*************
```

Phylogenetic tree (CLUSTAL O, v1.2.4)

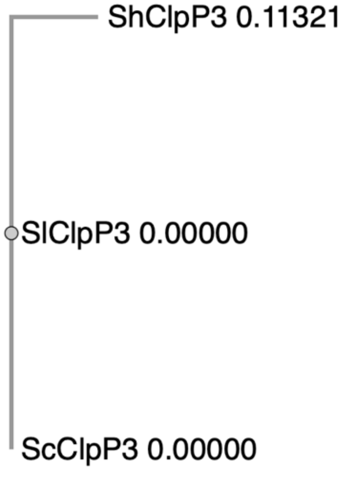

Of note, *Streptomyces griseus subsp. griseus* ATCC 12648 does not encode ClpP3, ClpP4 and ClpP5.

D

ClpP4 Multiple sequence alignment (CLUSTAL O, v1.2.4, EMBL-EBI)

```
ShClpP4      MGTYTIPNVIERTPQGERSFDVFSRLLNERIIFLGTEIDDGVANVVIAQLLHLESSSPES      60
SlClpP4      MGSYTIPNVVERTPQGERSYDVFSRLLSERIIFLGTEIDDGVANVVIAQLLHLESSAPES      60
ScClpP4      MGSYTIPNVVERTPQGERSYDVFSRLLSERIIFLGTEIDDGVANVVIAQLLHLESSAPES      60
             **:******:*********:*******.****************************:***

ShClpP4      EIAIYLNSPGGSFTSLMAIYDTMTFVQAPISTFCVGQAASTAAVLLAGGDPGRRFVLEHS      120
SlClpP4      EIAVYINSPGGSFTSLMAIYDTMTFVQAPISTFCVGQAASTAAVLLAGGDPGRRFVLEHA      120
ScClpP4      EIAVYINSPGGSFTSLMAIYDTMTFVQAPISTFCVGQAASTAAVLLAGGDPGRRFVLEHA      120
             ***:*:*****************************************************:

ShClpP4      RVLLGQPAAGGQRGMVSDLALQAKEMVRIRSQVEEVLARHTHHDVPTLRADMDRDKVFTA      180
SlClpP4      RVLLGQPASGGRQGTVSDLALQAKEMVRIRSQVEEVLARHTHHDVATLRADMDRDKVFTA      180
ScClpP4      RVLLGQPASGGRQGTVSDLALQAKEMVRIRSQVEEVLARHTHHDVATLRADMDRDKVFTA      180
             ********:**::* ****************************** **************

ShClpP4      QEAVAYGLADEVLSRRLVKV      200
SlClpP4      QEAVAYGLADEVLARRLTRV      200
ScClpP4      QEAVAYGLADEVLARRLTRV      200
             *************:***.:*
```

Phylogenetic tree (CLUSTAL O, v1.2.4)

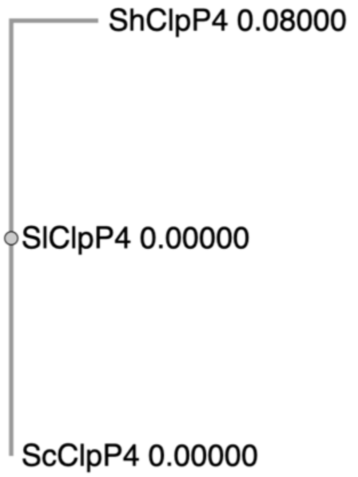

Of note, *Streptomyces griseus subsp. griseus* ATCC 12648 does not encode ClpP3, ClpP4 and ClpP5.

E

ClpP5 Multiple sequence alignment (CLUSTAL O, v1.2.4, EMBL-EBI)

```
ShClpP5      MTRPSARYVLPEFTERTGSGQRTMDPYSKLLEERIVFLGAPVDETSANDVMAQFMYLEHQ     60
SlClpP5      MTRPSARHVLPEFTERTSAGTRTSDPYSKLLQERIVFLGTPVDETSANDVTAQLMYLEHQ     60
ScClpP5      MTRPSARHVLPEFTERTSAGTRTSDPYSKLLQERIVFLGTPVDETSANDVTAQLMYLEHQ     60
             *******:*********.:* ** *******:*******:********** **:******

ShClpP5      APERDISLYINSPGGTFHAMTALYDTMRYVSCDVETICLGQAGAASSVLLAAGTPGKRFA     120
SlClpP5      APDRDIELYVNSPGGSFTAMTAIYDTMRYVACDVATTCLGQAGPSAAVLLAAGTPGKRAA     120
ScClpP5      APDRDIELYVNSPGGSFTAMTAIYDTMRYVACDVATTCLGQAGPSAAVLLAAGTPGKRAA     120
             **:***.**:*****:* ****:*******:*** * ****** :::*********** *

ShClpP5      LPDARLVIHQPALPEPVRGQASDLAIQADELTRIRGRMEEMLALHTGRTREQVSTDIERD     180
SlClpP5      LPGARVVLHQPALTEPVRGQAGDLAVHAAELVRVRARLEEILVRHTGRTPGQVAADLERD     180
ScClpP5      LPGARVVLHQPALTEPVRGQAGDLAVHAAELVRVRARLEEILVRHTGRTPGQVAADLERD     180
             **.**:*:***** *******.***::* **.*:*.*:**:*. *****  **::*:***

ShClpP5      KILTAQEAVEYGLVDGIIPSRKATLAPPTGR  211
SlClpP5      TVLDARQAREYGLVDRIVPGRRTPPASSGAR  211
ScClpP5      TVLDARQAREYGLVDRIVPGRRTPPASSGAR  211
             .:* *::* ****** *:*.*::  *   .*
```

Phylogenetic tree (CLUSTAL O, v1.2.4)

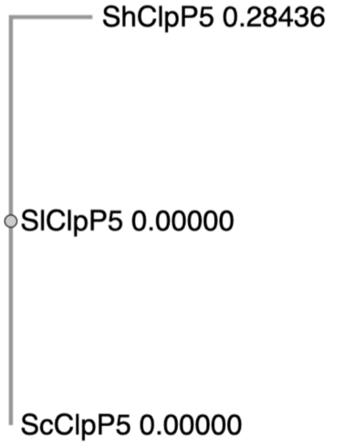

Of note, *Streptomyces griseus subsp. griseus* ATCC 12648 does not encode ClpP3, ClpP4 and ClpP5.

F

ShClpP Multiple sequence alignment (CLUSTAL O, v1.2.4, EMBL-EBI)

```
ShClpP4      --------------------------------MGTYTIPNVI-ERTPQGERSFDVFSRLLN      28
ShClpP2      MNQFPGSGIYDRMHAVQDMSASQGRYTGPQAESRYIIPRFV-ERTSQGIREYDPYAKLFE      59
ShClpP5      ----------------------------MTRPSARYVLPEFT-ERTGSGQRTMDPYSKLLE      32
ShClpP3      ------------MTPL-----------------TALAPRAEEGDTPPTRFDDQLAAQLLA      31
ShClpP1      -MRRPGAVVRRAGGYV-----------------TNLMPSAAGEPSIGGGLGDQVYNRLLN      42
ShClpADEP    --------MKD----I-----------------KELTGRT--LGASRWNLNDQVMHRLMD      29
                                                         :       :   :*:

ShClpP4      ERIIFLGTEIDDGVANVVIAQLLHLESSSPESEIAIYLNSPGGSFTSLMAIYDTMTFVQA      88
ShClpP2      ERVIFLGVQIDDASANDVMAQLLCLESMDPDRDISVYINSPGGSFTALTAIYDTMQYVKP      119
ShClpP5      ERIVFLGAPVDETSANDVMAQFMYLEHQAPERDISLYINSPGGTFHAMTALYDTMRYVSC      92
ShClpP3      QRIVLLGTQVDEVSANRVCSQLLILSAEDAHTDISLYINSPGGAVHAGLAIYDTMRLIPN      91
ShClpP1      ERIIFLGQPVDDDIANKITAQLLLLA-SDPEKDIYLYINSPGGSITAGMAIYDTMQYIKN      101
ShClpADEP    ERIIMLGQEVDDAGSNAICSQLLLLA-GDSPRDISLYINSPGGSVTAGMAIYDTMNYIEN      88
             :*:::**  :*:  :* : :*:: *       :* :*:*****:. :  *:****  :

ShClpP4      PISTFCVGQAASTAAVLLAGGDPGRRFVLEHSRVLLGQPA-AGGQRGMVSDLALQAKEMV      147
ShClpP2      DVQTVCMGQAASAAAVLLAAGTPGKRMALPNARVLIHQPY-SETGRGQVSDLEIAANEIL      178
ShClpP5      DVETICLGQAGAASSVLLAAGTPGKRFALPDARLVIHQPALPEPVRGQASDLAIQADELT      152
ShClpP3      DVSTLAMGFAASMGQFLLSVGAPGKRYALPNARIMMHQPS--AGIGGTTADIEIQAQNLE      149
ShClpP1      DVVTIAMGLAASMGQFLLSAGTPGKRFALPNAEILIHQPS--AGLAGSASDIKIHAERLL      159
ShClpADEP    DVVTVAMGTAASMGQFLLTAGTPGKRIVLPHAEILMHQPS--AGLGGSASDIKIHAERLI      146
              : *..:* *.: . .**: * **:* .* .:.::  **       * .:*: : *..:

ShClpP4      RIRSQVEEVLARHTHHDVPTLRADMDRDKVFTAQEAVAYGLADEVLSRRLVKV-------------200
ShClpP2      RMRSQLEDMLAKHSTTPVEKIREDIERDKILTAEDALSYGLIDQVISTRKMDNSSLR---------235
ShClpP5      RIRGRMEEMLALHTGRTREQVSTDIERDKILTAQEAVEYGLVDGIIPSRKATLAPPTGR-------211
ShClpP3      FTKRTIERITAEHTGQSPENISRDGDRDRWFTAEEAREYGMVDRVVESLSD-VRPAATRRRMGLQ-213
ShClpP1      HTKKRMAELTSQHTGQTIEQITRDSDRDRWFDAFEAKEYGLIDDVMTTAAG-MPGGGGTGA-----219
ShClpADEP    RVKKRMIDITAQHTGRTVEEIKRDSDRDRWFSADEAVEYGLADRVEYVAAT-VPGNIGAAK-----206
               :  :  : : *:      :  * :**: : * :*  **: * :
```

Phylogenetic tree (CLUSTAL O, v1.2.4)

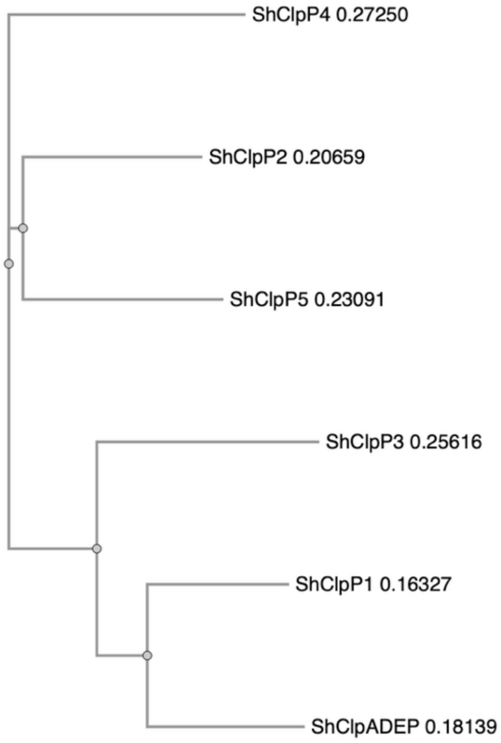

Supplement: Fig. S1 — Multiple amino acid sequence alignment and phylogenetic tree analyses of ClpP proteins from Streptomyces species. [file mbio.01652-25-s0001.pdf]
