# Supplementary material for: Self-resistance mechanism to acyldepsipeptide antibiotics in the Streptomyces producer
Source: mBio. 2025 Oct 6;16(11):e01652-25. doi: 10.1128/mbio.01652-25 (PMC12607617; doi:10.1128/mbio.01652-25)
Supplement: Fig. S2 — Relative transcript levels of clpP1-P5 and clpPADEP in S. hawaiiensis. [file mbio.01652-25-s0002.pdf]

SI file

Self-resistance mechanism to acyldepsipeptide antibiotics in the *Streptomyces* producer

Dhana Thomy[1,2,4], Laura Reinhardt[1,2,4], Elisa Liebhart[1,2], Mirita Franz-Wachtel[2,3], Boris Maček[2,3], Peter Sass[1,2+], Heike Brötz-Oesterhelt[1,2*+].
[1]Department of Microbial Bioactive Compounds, IMIT, University of Tübingen, Germany. [2]Cluster of Excellence - Controlling Microbes to Fight Infections, University of Tübingen, Germany. [3]Proteome Center Tübingen, University of Tübingen, Germany. [4]Dhana Thomy and Laura Reinhardt contributed equally to this work. Author order was determined by seniority. *heike.broetz-oesterhelt@uni-tuebingen.de.
[+]Peter Sass and Heike Brötz-Oesterhelt share senior authorship.

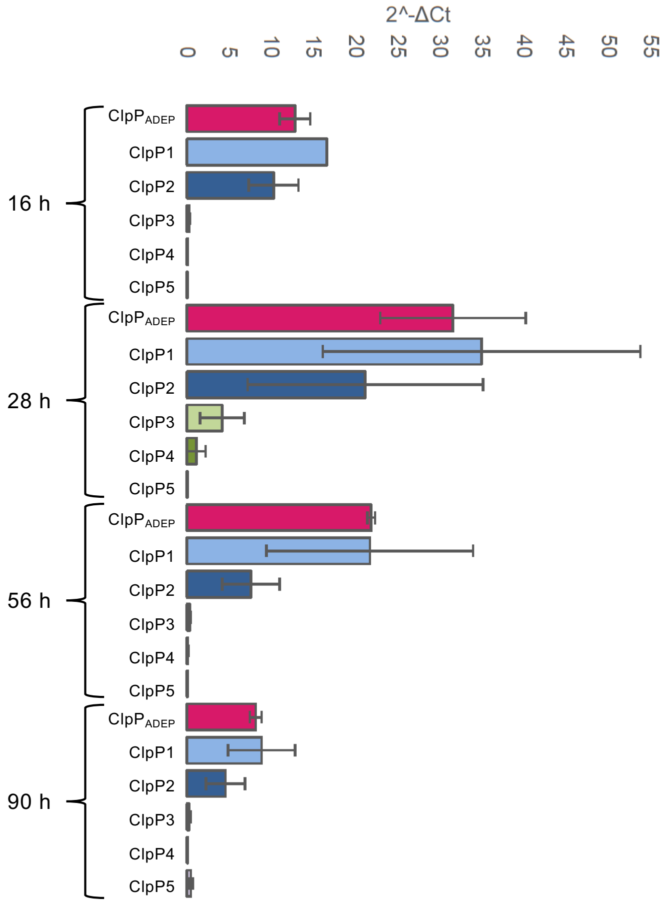

Figure S2. Relative transcript levels of *clpP1-P5* and *clpP$_{ADEP}$* in *S. hawaiiensis*. qPCR analysis reveals that *clpP$_{ADEP}$* is expressed already in early growth phase (time point: 16 h). Relative expression levels of the *clpP* genes in *S. hawaiiensis* at different growth stages. The mean values of two biological replicates, each with two technical replicates, are shown, as well as the maximum deviation from the mean.
