# Supplementary material for: Self-resistance mechanism to acyldepsipeptide antibiotics in the Streptomyces producer
Source: mBio. 2025 Oct 6;16(11):e01652-25. doi: 10.1128/mbio.01652-25 (PMC12607617; doi:10.1128/mbio.01652-25)
Supplement: Fig. S3 — Quantitative proteomic analysis of ClpP1, ClpP2 and ClpPADEP in S. hawaiiensis and S. lividans. [file mbio.01652-25-s0003.pdf]

SI file

Self-resistance mechanism to acyldepsipeptide antibiotics in the *Streptomyces* producer

Dhana Thomy[1,2,4], Laura Reinhardt[1,2,4], Elisa Liebhart[1,2], Mirita Franz-Wachtel[2,3], Boris Maček[2,3], Peter Sass[1,2+], Heike Brötz-Oesterhelt[1,2*+].
[1]Department of Microbial Bioactive Compounds, IMIT, University of Tübingen, Germany. [2]Cluster of Excellence - Controlling Microbes to Fight Infections, University of Tübingen, Germany. [3]Proteome Center Tübingen, University of Tübingen, Germany. [4]Dhana Thomy and Laura Reinhardt contributed equally to this work. Author order was determined by seniority. *heike.broetz-oesterhelt@uni-tuebingen.de.
[+]Peter Sass and Heike Brötz-Oesterhelt share senior authorship.

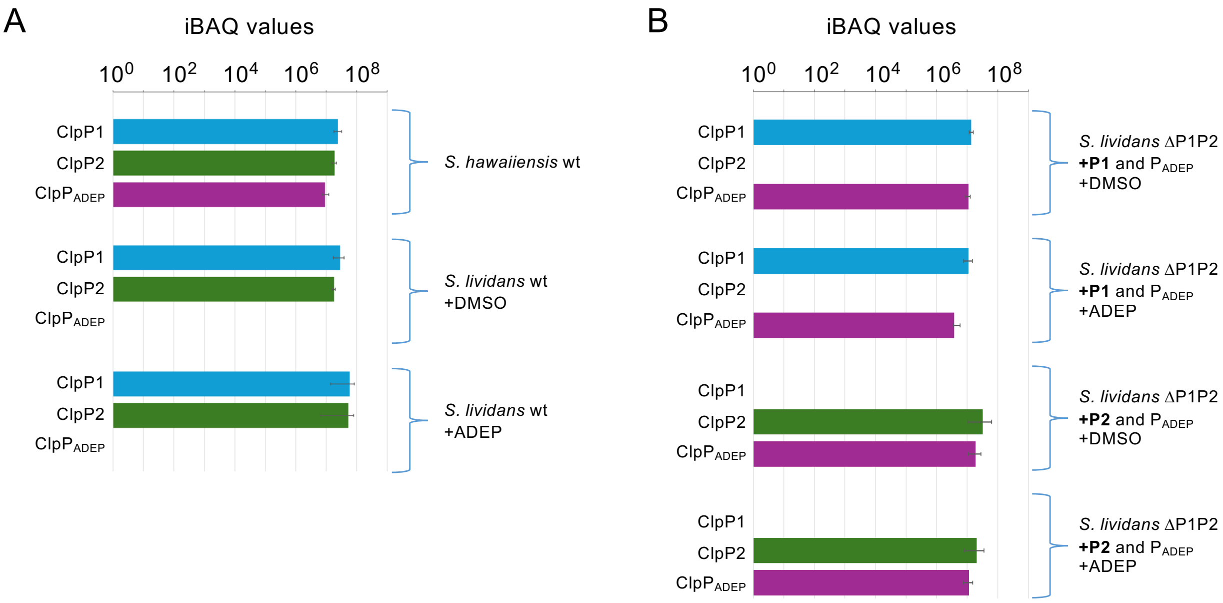

C

		iBAC values			
		replicate 1	replicate 2	replicate 3	Mean
S. hawaiiensis WT	ClpP1	18039000	32123000	22890000	24350667
	ClpP2	21660000	19237000	15323000	18740000
	ClpPAdep	7334300	12108000	8090300	9177533
S. lividans WT DMSO	ClpP1	30060000	39016000	17172000	28749333
	ClpP2	18656000	19728000	15849000	18077667
	ClpPAdep	0	0	0	0
S. lividans WT ADEP	ClpP1	13871000	79723000	83375000	58989667
	ClpP2	6704500	79446000	72490000	52880167
	ClpPAdep	0	0	0	0
S. lividans ΔP1P2, ClpP1ClpPA DMSO	ClpP1	11551000	15777000	13433000	13587000
	ClpP2	0	0	0	0
	ClpPAdep	12682000	11694000	8799300	11058433
S. lividans ΔP1P2, ClpP1ClpPA ADEP	ClpP1	10424000	14749000	7588900	10920633
	ClpP2	0	0	0	0
	ClpPAdep	3544900	5857200	1965000	3789033
S. lividans ΔP1P2, ClpP2ClpPA DMSO	ClpP1	0	0	0	0
	ClpP2	10402000	63082000	24185000	32556333
	ClpPAdep	10919000	28115000	17979000	19004333
S. lividans ΔP1P2, ClpP2ClpPA ADEP	ClpP1	0	0	0	0
	ClpP2	8048300	17952000	35375000	20458433
	ClpPAdep	7368500	11861000	15073000	11434167

Figure S3. Quantitative proteomic analysis of ClpP1, ClpP2 and ClpP$_{ADEP}$ in *S. hawaiiensis* and *S. lividans*. A-C. Intensity-based absolute quantification (iBAQ). **D-F.** Label-free quantification (LFQ). Quantitative proteomics show that protein levels of ClpP$_{ADEP}$ were in a similar range to those of ClpP1 and ClpP2, indicating the presence of considerable amounts of ClpP$_{ADEP}$. Mean iBAQ and LFQ values from three biological replicates are shown. Error bars indicate the difference between the lowest and highest values.

D

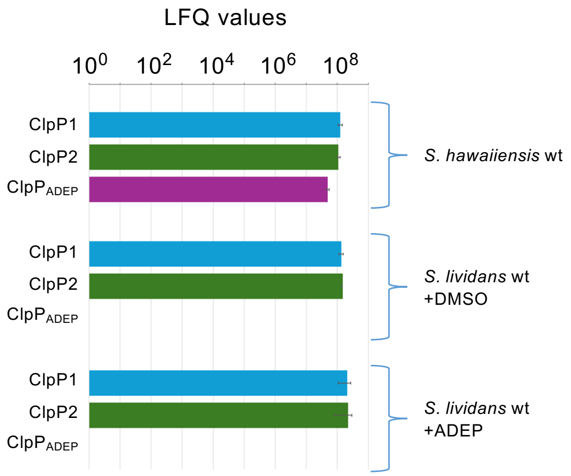

E

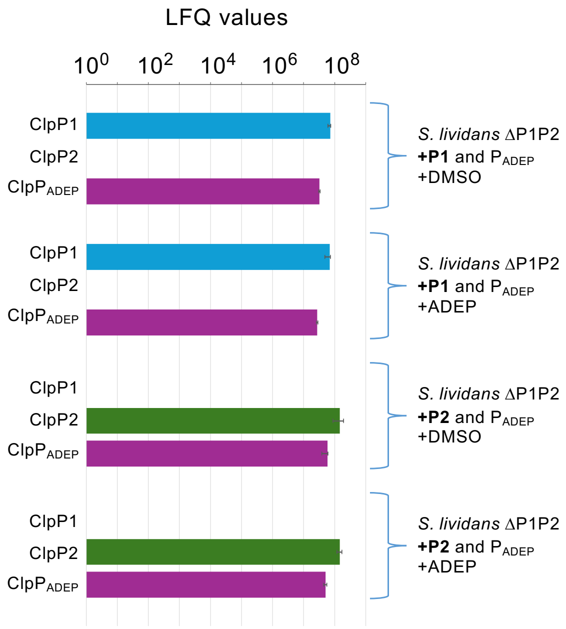

F

		LFQ values			
		replicate 1	replicate 2	replicate 3	Mean
S. hawaiiensis WT	ClpP1	106320000	144250000	122500000	124356667
	ClpP2	123710000	96530000	102580000	107606667
	ClpPAdep	43736000	55281000	47881000	48966000
S. lividans WT DMSO	ClpP1	135000000	156030000	113890000	134973333
	ClpP2	175050000	137460000	136820000	149776667
	ClpPAdep	0	0	0	0
S. lividans WT ADEP	ClpP1	105540000	248020000	272480000	208680000
	ClpP2	80529000	295710000	284580000	220273000
	ClpPAdep	0	0	0	0
S. lividans ΔP1P2, ClpP1ClpPA DMSO	ClpP1	61177000	74683000	83319000	73059667
	ClpP2	0	0	0	0
	ClpPAdep	33840000	31499000	30761000	32033333
S. lividans ΔP1P2, ClpP1ClpPA ADEP	ClpP1	86212000	73616000	48662000	69496667
	ClpP2	0	0	0	0
	ClpPAdep	26140000	28731000	26978000	27283000
S. lividans ΔP1P2, ClpP2ClpPA DMSO	ClpP1	0	0	0	0
	ClpP2	88665000	195220000	151280000	145055000
	ClpPAdep	38359000	60577000	77354000	58763333
S. lividans ΔP1P2, ClpP2ClpPA ADEP	ClpP1	0	0	0	0
	ClpP2	118460000	151410000	172140000	147336667
	ClpPAdep	44071000	53829000	55590000	51163333
