# Supplementary material for: Self-resistance mechanism to acyldepsipeptide antibiotics in the Streptomyces producer
Source: mBio. 2025 Oct 6;16(11):e01652-25. doi: 10.1128/mbio.01652-25 (PMC12607617; doi:10.1128/mbio.01652-25)
Supplement: Fig. S4 — Size exclusion chromatography of ShClpPADEP. [file mbio.01652-25-s0004.pdf]

SI file

Self-resistance mechanism to acyldepsipeptide antibiotics in the *Streptomyces* producer

Dhana Thomy[1,2,4], Laura Reinhardt[1,2,4], Elisa Liebhart[1,2], Mirita Franz-Wachtel[2,3], Boris Maček[2,3], Peter Sass[1,2+], Heike Brötz-Oesterhelt[1,2*+].
[1]Department of Microbial Bioactive Compounds, IMIT, University of Tübingen, Germany. [2]Cluster of Excellence - Controlling Microbes to Fight Infections, University of Tübingen, Germany. [3]Proteome Center Tübingen, University of Tübingen, Germany. [4]Dhana Thomy and Laura Reinhardt contributed equally to this work. Author order was determined by seniority. *heike.broetz-oesterhelt@uni-tuebingen.de.
[+]Peter Sass and Heike Brötz-Oesterhelt share senior authorship.

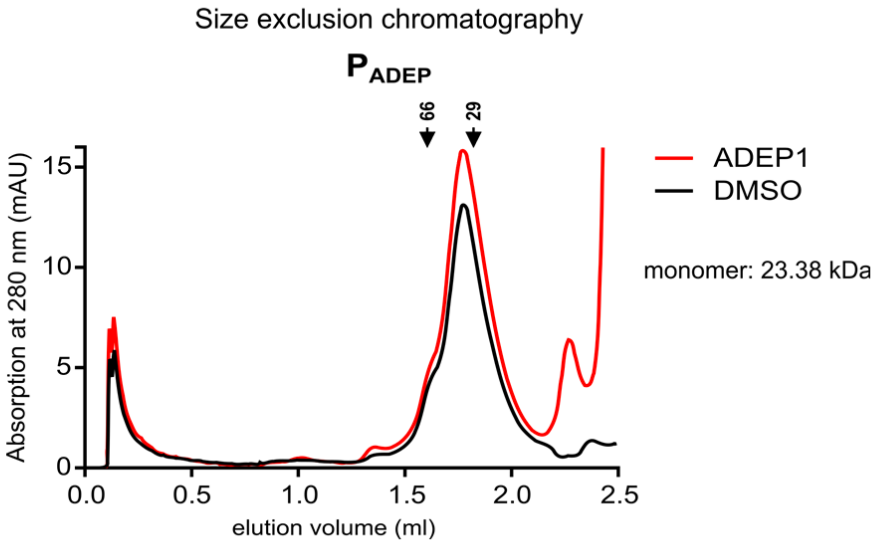

Figure S4. Size exclusion chromatography of ShClpP$_{ADEP}$. ClpP$_{ADEP}$ alone eluted mostly in lower oligomeric states. The oligomeric behavior of ShClpP$_{ADEP}$ (50 µM) was analyzed in the absence (black curve) and presence (red curve) of ADEP1 (80 µM) using the SuperdexTM 200 Increase 3.2 300 column. The experiment was performed in two biological replicates.
