# Supplementary material for: Self-resistance mechanism to acyldepsipeptide antibiotics in the Streptomyces producer
Source: mBio. 2025 Oct 6;16(11):e01652-25. doi: 10.1128/mbio.01652-25 (PMC12607617; doi:10.1128/mbio.01652-25)
Supplement: Fig. S6 — Pull-down experiment of shClpP1 and ClpPADEP-His6. [file mbio.01652-25-s0006.pdf]

SI file

Self-resistance mechanism to acyldepsipeptide antibiotics in the *Streptomyces* producer

Dhana Thomy[1,2,4], Laura Reinhardt[1,2,4], Elisa Liebhart[1,2], Mirita Franz-Wachtel[2,3], Boris Maček[2,3], Peter Sass[1,2+], Heike Brötz-Oesterhelt[1,2*+].
[1]Department of Microbial Bioactive Compounds, IMIT, University of Tübingen, Germany. [2]Cluster of Excellence - Controlling Microbes to Fight Infections, University of Tübingen, Germany. [3]Proteome Center Tübingen, University of Tübingen, Germany. [4]Dhana Thomy and Laura Reinhardt contributed equally to this work. Author order was determined by seniority. *heike.broetz-oesterhelt@uni-tuebingen.de.
[+]Peter Sass and Heike Brötz-Oesterhelt share senior authorship.

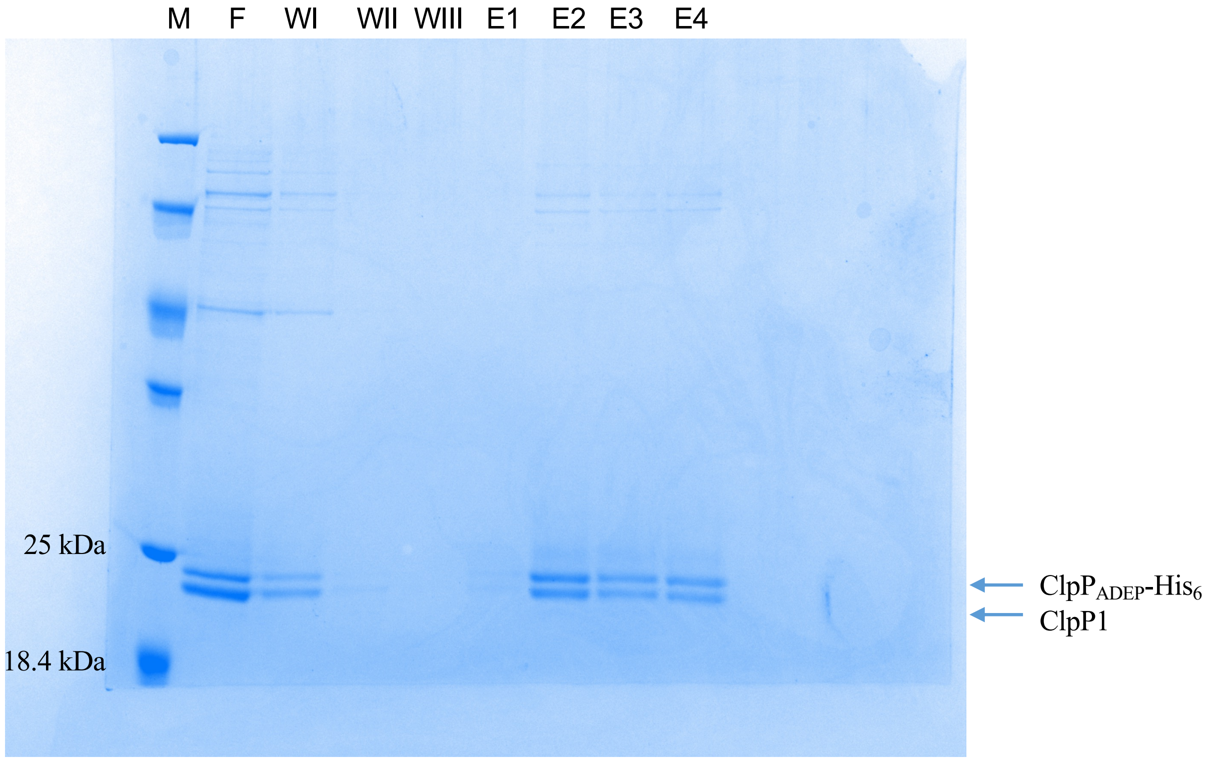

Figure S6. Pull-down experiment of shClpP1 and ClpP$_{ADEP}$-His$_6$. Pull-down experiment applying pre-incubated mixtures of untagged shClpP1 and ClpP$_{ADEP}$-His$_6$ to nickel-nitrilotriacetic acid (Ni-NTA) agarose. SDS-PAGE of flow-through (F), wash steps (W I to III) and elution fractions (E 1 to 4), M, protein marker. SDS-PAGE shows co-elution of both shClpP1 and ClpP$_{ADEP}$-His$_6$ in a similar ratio, indicating a direct interaction of both homologs.
