# Supplementary material for: Self-resistance mechanism to acyldepsipeptide antibiotics in the Streptomyces producer
Source: mBio. 2025 Oct 6;16(11):e01652-25. doi: 10.1128/mbio.01652-25 (PMC12607617; doi:10.1128/mbio.01652-25)
Supplement: Fig. S7 — Protein degradation assays: ClgR/PopR. [file mbio.01652-25-s0007.pdf]

SI file

Self-resistance mechanism to acyldepsipeptide antibiotics in the *Streptomyces* producer

Dhana Thomy[1,2,4], Laura Reinhardt[1,2,4], Elisa Liebhart[1,2], Mirita Franz-Wachtel[2,3], Boris Maček[2,3], Peter Sass[1,2+], Heike Brötz-Oesterhelt[1,2*+].
[1]Department of Microbial Bioactive Compounds, IMIT, University of Tübingen, Germany. [2]Cluster of Excellence - Controlling Microbes to Fight Infections, University of Tübingen, Germany. [3]Proteome Center Tübingen, University of Tübingen, Germany. [4]Dhana Thomy and Laura Reinhardt contributed equally to this work. Author order was determined by seniority. *heike.broetz-oesterhelt@uni-tuebingen.de.
[+]Peter Sass and Heike Brötz-Oesterhelt share senior authorship.

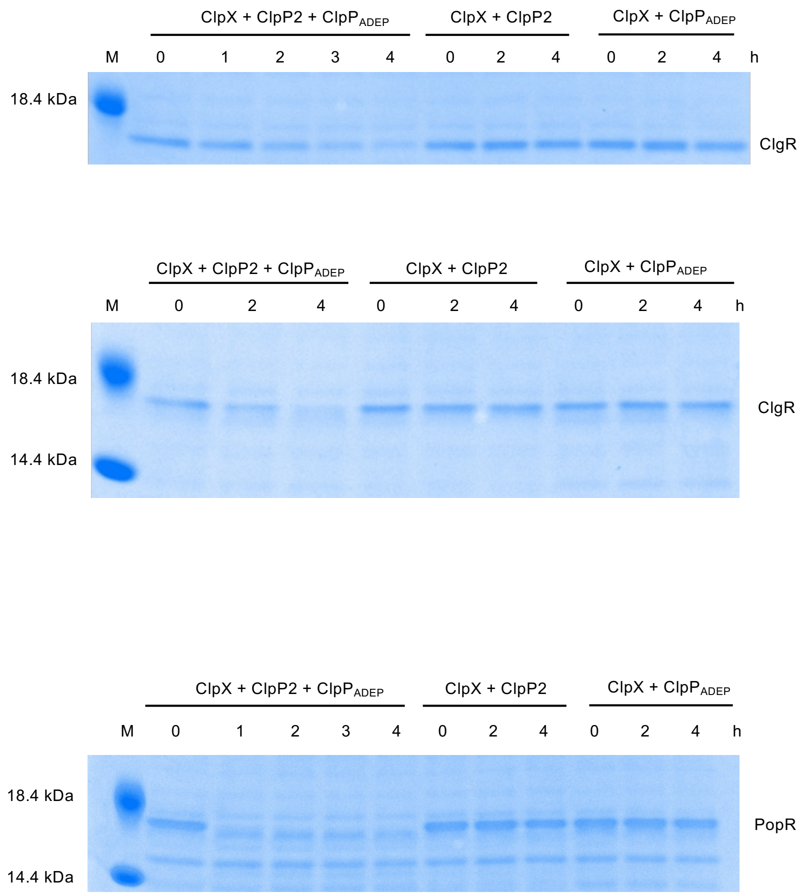

Figure S7. Protein degradation assays. *In vitro* protease activity assays using heterologously expressed and isolated *S. hawaiiensis* proteins ClpP$_{ADEP}$ and ClpP2, the Clp-ATPase protein ClpX, as well as the natural ClpXP2P1 substrates ClgR and PopR. Neither ClpP2 nor ClpP$_{ADEP}$ alone are capable of protein degradation with ClpX. However, when ClpP$_{ADEP}$, ClpP2 and ClpX were combined, ClgR and PopR were digested, thus validating that ClpP$_{ADEP}$ and ClpP2 jointly substitute for the ClpP1P2 core in natural protein degradation.
