# Supplementary material for: Self-resistance mechanism to acyldepsipeptide antibiotics in the Streptomyces producer
Source: mBio. 2025 Oct 6;16(11):e01652-25. doi: 10.1128/mbio.01652-25 (PMC12607617; doi:10.1128/mbio.01652-25)
Supplement: Fig. S8 — Protein degradation assays: casein. [file mbio.01652-25-s0008.pdf]

SI file

Self-resistance mechanism to acyldepsipeptide antibiotics in the *Streptomyces* producer

Dhana Thomy[1,2,4], Laura Reinhardt[1,2,4], Elisa Liebhart[1,2], Mirita Franz-Wachtel[2,3], Boris Maček[2,3], Peter Sass[1,2+], Heike Brötz-Oesterhelt[1,2*+].
[1]Department of Microbial Bioactive Compounds, IMIT, University of Tübingen, Germany. [2]Cluster of Excellence - Controlling Microbes to Fight Infections, University of Tübingen, Germany. [3]Proteome Center Tübingen, University of Tübingen, Germany. [4]Dhana Thomy and Laura Reinhardt contributed equally to this work. Author order was determined by seniority. *heike.broetz-oesterhelt@uni-tuebingen.de.
[+]Peter Sass and Heike Brötz-Oesterhelt share senior authorship.

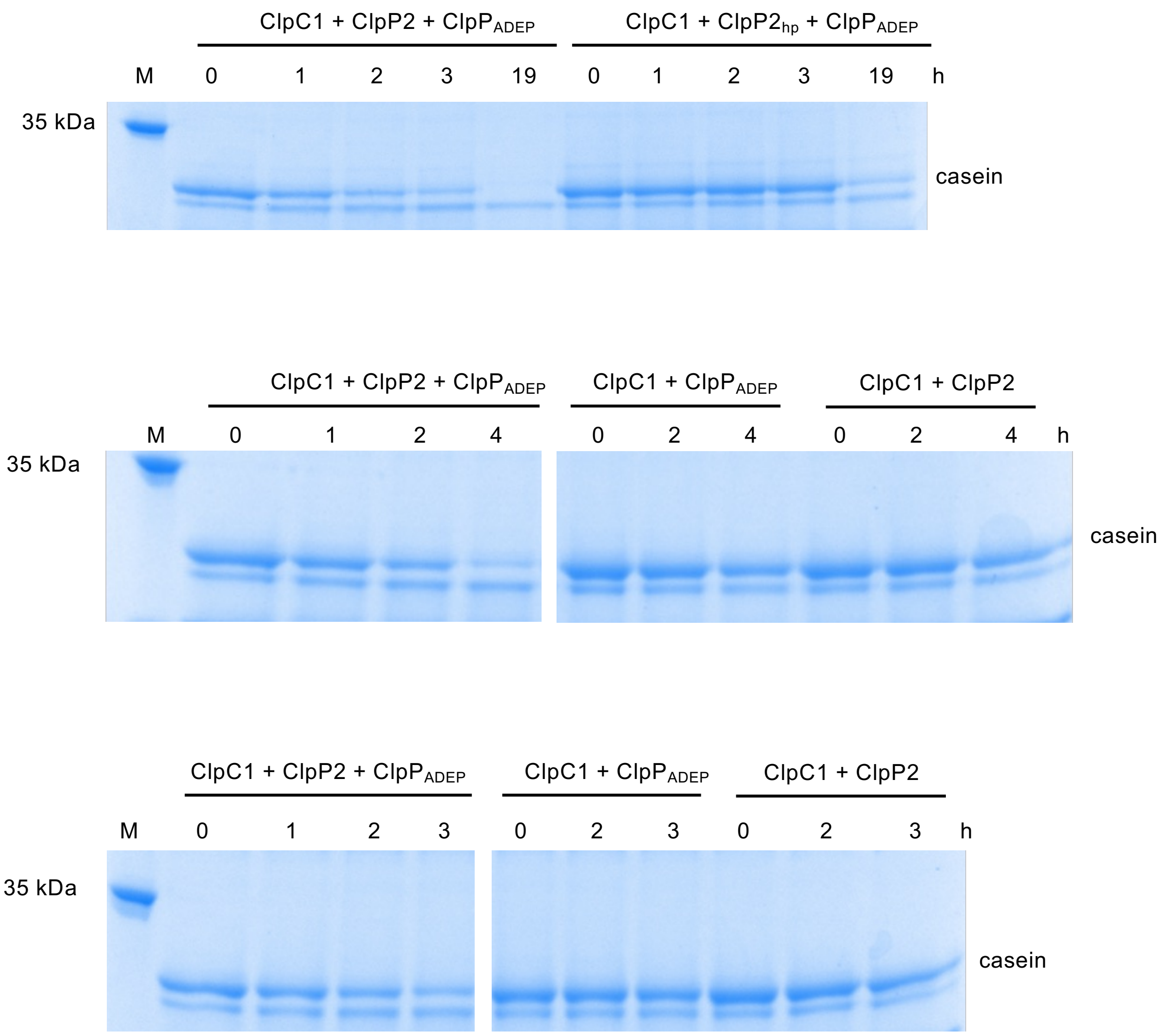

Figure S8. Protein degradation assays. *In vitro* protease activity assays using the isolated *S. hawaiiensis* proteins $ClpP_{ADEP}$ and ClpP2, the Clp-ATPase protein ClpC1, as well as the model protein substrate ß-casein. In addition, a ClpP2 hydrophobic pocket mutant ($ClpP2_{hp}$) was used to analyze Clp-ATPase binding. When the Clp-ATPase ClpC1 is used in *in vitro* degradation assays with the model protein substrate ß-casein, proteolysis occurs only when $ClpP_{ADEP}$, ClpP2 and ClpC1 are combined.
