# Supplementary material for: Self-resistance mechanism to acyldepsipeptide antibiotics in the Streptomyces producer
Source: mBio. 2025 Oct 6;16(11):e01652-25. doi: 10.1128/mbio.01652-25 (PMC12607617; doi:10.1128/mbio.01652-25)
Supplement: Supplemental text — Supplemental methods. [file mbio.01652-25-s0009.pdf]

SI file

Self-resistance mechanism to acyldepsipeptide antibiotics in the *Streptomyces* producer

Dhana Thomy[1,2,4], Laura Reinhardt[1,2,4], Elisa Liebhart[1,2], Mirita Franz-Wachtel[2,3], Boris Maček[2,3], Peter Sass[1,2+], Heike Brötz-Oesterhelt[1,2*+].
[1]Department of Microbial Bioactive Compounds, IMIT, University of Tübingen, Germany. [2]Cluster of Excellence - Controlling Microbes to Fight Infections, University of Tübingen, Germany. [3]Proteome Center Tübingen, University of Tübingen, Germany. [4]Dhana Thomy and Laura Reinhardt contributed equally to this work. Author order was determined by seniority. *heike.broetz-oesterhelt@uni-tuebingen.de.
[+]Peter Sass and Heike Brötz-Oesterhelt share senior authorship.

Supplemental Methods

Bacterial strains, culture conditions and plasmids

All strains used in this study are listed and referenced in table S1. *Streptomyces hawaiiensis* NRRL 15010 is publicly available from the Agricultural Research Service Culture Collection (NRRL). *S. lividans* TK24, *S. coelicolor* A3(2) and *S. griseus* Waksman were kindly provided by Dr. Günther Muth (University of Tübingen, Germany). *Streptomyces* wild type strains and mutants were grown at 30 °C on MS-$MgCl_2$ agar (2% soy flour, 2% mannitol, 2% agar, 10 mM $MgCl_2$) or in Tryptic Soy Broth (TSB) with apramycin (50 µg/ml) and/or hygromycin B (50 µg/ml) as appropriate.

E. coli DH5α, *E. coli* SG1146a (BL21(DE3) *clpP::cam*), and *E. coli* ET12567 (pUB307 or pUZ8002 *neo::bla*) strains were propagated in lysogeny broth (LB) with ampicillin (100 µg/ml), apramycin (50 µg/ml), hygromycin B (50 µg/ml), kanamycin (50 µg/ml), and/or chloramphenicol (30 µg/ml) as required. The pSET152ermE*ΔHindIII plasmid (gift from Prof. Dr. Till Schäberle, University of Giessen, Germany) was used to clone and heterologously express *clpP*$_{ADEP}$. The plasmids pIJ12551 and pIJ10257 used for the complementation of *S. lividans* Δ*clpP* mutants with different *clpP* genes were kindly provided by Prof. Dr. Marc Buttner (John Innes Center, Norwich, UK). Plasmids for the expression of *clp*, *clgR* and *popR* genes of *S. hawaiiensis* NRRL 15010 were obtained by ligating PCR-amplified gene sequences with linearized pET11a or pET22b vectors (Merck-Novagen), respectively. As PCR template, genomic DNA of *S. hawaiiensis* NRRL 15010 (NCBI Genbank accession number: CP021978.1) was used to amplify *shclpP1* (orf CEB94_14110), *shclpP2* (orf CEB94_14105), *shClpX* (orf CEB94_14100), *shclpC1* (orf CEB94_23085), *shclgR* (orf CEB94_30145), and *shpopR* (MT943519). All constructs used in this study were generated by traditional cloning. DNA fragments of the genes of interest were amplified using Q5 High-Fidelity DNA Polymerase (NEB) and the respective primer pair and cloned into the respective vector via restriction-ligation utilizing *E. coli* JM109 or *E. coli* DH5α as cloning strains. All constructs were verified by Sanger sequencing (LGC Genomics). *Streptomyces* vectors were conjugated into the respective *Streptomyces* strains via *E. coli* ET12567 pUB307 or pUZ8002 as described elsewhere (1). *E. coli* vectors were introduced into chemo-competent the *E. coli* SG1146a Δ*clpP* deletion strain (2) for protein expression.

Site-directed mutagenesis

Constructs for the expression of functionally altered ClpP homologs were generated via site-directed mutagenesis as described in the instruction manual for the QuickChange Lightning Site-Directed Mutagenesis Kit (Agilent Technologies) using the ReproFast-DNA polymerase (Genaxxon) and *E. coli* JM109 or *E. coli* DH5α as cloning strains.

Generation of *clpP* deletion mutants

To achieve markerless gene knockouts, we amplified 1.5 kb long flanking regions of the respective gene via PCR with the Q5 High-Fidelity DNA Polymerase (NEB) and integrated them into the knockout vector pGM-GUS-Xba by Gibson assembly using the NEBuilder HiFi DNA Assembly Master Mix (NEB) and *E.*

coli JM109 or *E. coli* DH5α as cloning strains. After verification by Sanger sequencing, the knockout constructs were transformed into *E. coli* ET12567 pUB307 and conjugated into *S. lividans* TK24 as described previously (1). Mutant colonies were grown with apramycin at 39 °C in TSB liquid cultures and subsequently on MS agar to maintain cells with integrated plasmids only. Spores were harvested and dilutions were plated on LB agar with 2 µg/ml of ADEP1 as selective pressure for plasmid loss. The obtained colonies were overlaid with 1 ml of an X-Gluc (5-Bromo-4-chloro-3-indolyl-β-D-glucuronide) solution in water (1 mg/ml) for a blue-white screen. For white colonies, loss of the vector and the respective *clpP* gene were further verified by colony PCR and Sanger sequencing of the PCR products.

ADEP sensitivity assays

Spore suspensions of respective *Streptomyces* strains were plated on Nutrient Extract (NE) agar (1% glucose, 0.2% yeast extract, 0.2% casamino acids, 0.1% Lab-Lemco Powder, pH 7.0). Paper disks containing 10 µg of ADEP1 were added to the plates, which were subsequently incubated at 30 °C for 2 days.

Preparation of protein extracts

The respective *Streptomyces* strains were grown in 10 ml of TSB medium with apramycin and/or hygromycin as appropriate for 40 h at 30 °C and 180 rpm. The mycelium was harvested and resuspended in 500 µl of lysis buffer (20 mM Tris, 5 mM EDTA disodium salt, 1 mM 2-mercaptoethanol; one cOmplete Mini, EDTA-free protease inhibitor cocktail tablet (Roche) per 10 ml of lysis buffer) in 2 ml lysis tubes. Following cell disruption using a Precellys homogenizer (Precellys evolution, Bertin technologies), cell debris was removed by two centrifugation steps (14,000 rpm, 10 min, 4 °C and 14,000 rpm, 20 mins, 4 °C) and the concentration of the protein extracts was determined by measuring the absorption at 280 nm with the NanoDrop 2000c spectrophotometer (Thermo Scientific).

Protein purification

For the expression and purification of Clp proteins and the substrates ClgR and PopR, the respective expression plasmids were transformed into the Δ*clpP* deletion strain *E. coli* SG1146a (72). Expression strains were utilized to inoculate cultures using LB or terrific broth (TB) respectively, supplemented with ampicillin (100 µg/ml). Cultures were grown with constant shaking at 37 °C until a mid-log exponential growth-phase of OD_{600}: 0.5 – 0.6 was reached and protein expression was induced with 0.1 – 1 mM IPTG. Expression cultures for ClpP1, the Clp-ATPases and PopR were further incubated for 4 - 5 h at 30 °C with constant shaking, whereas ClpP2, $ClpP_{ADEP}$ and ClgR expression was carried out for 16 h at 30 °C and 20 °C, respectively. Next, cells were harvested by centrifugation. The following purification procedure was performed at 4 °C. For cell disruption, glass beads were used (150-212 µm) in a Precellys homogenizer (Precellys evolution, Bertin technologies). Cell lysates were centrifuged, and supernatants were passed through 0.45 µm membrane filters (Sarstedt) to remove cell debris before further processing. For purification of untagged ClpP1, the respective supernatants were applied to 1 ml HiTrap™ Q XL columns (GE Healthcare) for anion exchange chromatography using the ÄKTA Start chromatography system (GE Healthcare) with buffer A (50 mM Tris, pH 8) and buffer B (50 mM Tris, 1 M NaCl, pH 8). For purification of His_6-tagged proteins, 500 - 700 µl Ni-NTA resin (Thermo-Fisher) was added to the supernatant and the suspension was incubated at 4 °C for at least 2 h. Affinity chromatography was performed using lysis buffer (50 mM NaH_2PO_4, 300 mM NaCl, 10 mM imidazole pH 7.7), wash buffer (50 mM NaH_2PO_4, 300 mM NaCl, 20 mM imidazole, pH 7.7), and elution buffer (50 mM NaH_2PO_4, 300 mM NaCl, 500 mM imidazole

pH 7.7). Protein fractions were pooled for buffer exchange employing a PD-10 Sephadex^{TM} G-25 desalting column (GE Healthcare) and centrifugal filters (Amicon®Ultracel®-10 K/30 K; Merck). Protein concentration and purity was determined via SDS PAGE analysis, Bradford assay (Bio-Rad, applying BSA as reference).

***In vitro* protein degradation assays**
For the degradation of casein in the presence of ADEP1 (30 µM), 10 µM ß-casein was used in combination with 2 µM ClpP1, 2 µM ClpP2 and 2 µM $ClpP_{ADEP}$ and incubated at 30 °C in activity buffer. Samples were taken at indicated time points and analyzed via SDS-PAGE. Experiments were performed at least in triplicates and representative SDS-PAGE images are shown. For the degradation of the two natural substrates ClgR and PopR by $ClpXP_{ADEP}P2$, experiments were conducted in 100 µl reaction volumes in activity buffer, adding 2 mM DTT at 30 °C. 5 µM $ClpP_{ADEP}$, 5 µM ClpP2, or 2.5 µM + 2.5 µM $ClpP_{ADEP}P2$ were combined with 2.5 µM ClpX to degrade ClgR or PopR (2 µM each). For the degradation of ß-casein (10 µM), 5 µM $ClpP_{ADEP}$, 5 µM ClpP2 or 2.5 µM + 2.5 µM $ClpP_{ADEP}P2$ were combined with 5 µM ClpC1 and incubated at 30 °C. Samples were taken at indicated time points. Experiments were conducted by at least using two biological replicates, and representative SDS-PAGE images are shown.

SDS-PAGE and immunoblotting
Protein samples were mixed with Noxex 4x Bolt LDS sample buffer (Invitrogen) at a ratio of 2:1, heated at 70 °C for 10 mins, and then applied to Bolt 12% Bis-Tris Plus gels (Invitrogen), followed by protein separation via SDS polyacrylamide gel electrophoresis using Bolt MOPS SDS running buffer. Gels were either stained with InstantBlue Coomassie protein stain (Sigma) to visualize protein bands or used for immunoblotting experiments. Therefore, proteins from gels were transferred to an Amersham Protean Premium Western blotting membrane with a pore size of 0.45 µm (GE Healthcare) via semi-dry Western blotting using the following transfer buffer: 25 mM Tris, 192 mM Glycine, 15% v/v methanol, 0.5% w/v SDS. Immobilized proteins were treated with primary mouse anti-His_6 (1:2000, IBA), anti-*Streptomyces* ClpP1 (1:1000, Philippe Mazodier) or anti-Streptomyces ClpP3 (1:1000, Philippe Mazodier) antibodies followed by polyclonal HRP conjugated rabbit anti-mouse IgG (1:2000, IBA) or polyclonal HRP conjugated goat anti-rabbit IgG (1:5000, Pierce). Chemiluminescent signals were detected after applying the ECL Prime Western blotting detection reagent (GE Healthcare) with a ChemiDoc documentation system (BioRad).

Analytical size exclusion chromatography
To analyze the oligomeric behavior of ClpP1 and $ClpP_{ADEP}$, the Superdex^{TM} 200 Increase 3.2/300 column (GE Healthcare) was used. 80 µM of each protein were preincubated at 4 °C for 24 h. Then, 320 µM ADEP1 was added to the samples and incubated for 30 min at 30 °C. As a control, $ClpP_{ADEP}P1$ was applied to the gel filtration column in the absence of ADEP1, after a preincubation time for 24 h at 4 °C. Additionally, $ClpP_{ADEP}P1$ samples were preincubated at 4°C for 2 h and 7 h and subsequently applied to the gel filtration column. To induce the formation of ClpP1 homo-tetradecamers after ADEP1 addition (320 µM), ClpP1 (80 µM) alone was used with or without preincubation. The protein markers thyroglobulin (669 kDa), apoferritin (443 kDa), ß-amylase (200 kDa), alcohol dehydrogenase (150 kDa, yeast), BSA (66 kDa) and carbonic anhydrase (29 kDa, bovine) were employed for size estimation.

ClpP pull-down assay

8 µM native ClpP1 and 8 µM His6-tagged $ClpP_{ADEP}$ were incubated separately or combined at 4 °C for 1 h and loaded on a nickel ion affinity chromatography column. To this end, 500 µL reaction volumes were used. The column was washed three times, using 500 µl lysis (1st) and wash buffer (2nd and 3rd time) an eluted in four steps, consisting of 50 µl elution buffer, respectively. Three biological replicates were performed, and representative SDS-PAGE images are shown.

RNA preparation

RNA purification was performed from 2–10 ml of culture, depending on the growth phase, using the innuSPEED Bacteria/Fungi RNA Kit (Analytik Jena) according to the manufacturer's instructions, with the following deviations. Upon collection, samples were mixed with twice the volume of RNAprotect reagent in a 15 or 50 ml tube, incubated for 10 min at RT, and then pelleted by centrifugation for 15 min at 4 °C and 4,000 rpm. The supernatant was discarded, and the samples were stored at -80 °C until further use. To isolate the RNA, the samples were slowly thawed on ice and mixed with 200 µl of Lysis Buffer LR. The cell material was transferred to a lysis tube using an inoculating loop, and the remaining buffer was also transferred to the lysis tube using a pipette. Cell disruption was performed using the Precellys Evolution (3 x 23 s at 6,500 rpm, with a 10 s break each, three repetitions). Subsequently, another 250 µl of LR was added, and the samples were incubated and further processed according to the protocol. After the RNA bound to the column, an additional DNase digestion was performed using the innuPREP DNase I Digest Kit (Analytik Jena) according to the manufacturer's instructions. Finally, the RNA was eluted in 2 x 20 µl of RNase-free water, its concentration was determined using a NanoDrop spectrophotometer, and stored at -20 °C.

Quantitative real-time PCR (qPCR)

To analyze the expression of selected genes, the enzyme reverse transcriptase was used to transcribe the inherently much more unstable RNA into stable, single-stranded cDNA. This cDNA can then be used as a template in quantitative real-time PCR (qPCR), where the formation of DNA double strands can be monitored in real time using an intercalating fluorescent dye. After isolation of the RNA, 1,000 ng was subjected to a DNase digest to exclude contamination with genomic DNA. RNase-free DNase was used for the digestion according to the manufacturer's protocol. 5 µl of such a digestion could be used directly for cDNA synthesis. The AffinityScript Multiple Temperature cDNA Synthesis Kit (Agilent Technologies) was used for this purpose according to the manufacturer's instructions. To exclude contamination from any components, a noRT control (no reverse transcriptase) was always performed, as signals in this control sample during qPCR cannot originate from amplified cDNA and indicate foreign DNA or gDNA contamination. Successful cDNA production was verified by analytical PCR for comparison with the noRT control and the isolated RNA before and after the additional DNase digestion. If a positive result was obtained, the resulting cDNA was used for qPCR. The qPCR was performed using the Brilliant III Ultra-Fast SYBR Green QPCR Master Mix (Agilent Technologies) and the associated protocol. Primers were designed for the genes of interest, which, when tested with gDNA as a template, amplified a specific 150-250 bp product. Primers required in the assay were combined and mixed beforehand to avoid errors caused by pipetting small volumes. The assays were pipetted together in special 96-well qPCR plates with lids (Aria 96 Well Plates, Skirted, LP; Optical Cap, 8x Strip; both from Agilent Technologies). 1 µl of the primer mix was always added, followed by 2 µl of the respective template (cDNA, noRT control, or water for a no-template control). Then, 17 µl of a master mix containing all other components was added. Brief

centrifugation ensured that the entire volume of the mixture was bubble-free at the bottom of the 96-well plate.

In-gel digestion of proteins for proteomics

Proteins were precipitated with acetone-methanol at -20°C overnight, and washed pellets were resolved in digestion buffer (6 M urea, 2 M thiourea, 10 mM Tris, pH 8.0). Ten micrograms of proteins were loaded on a NuPAGE 12% Bis-Tris Gel (Thermo Fisher Scientific) for a short SDS based gel electrophoresis. Proteins were stained with colloidal Coomassie (ReadyBlue Protein Gel Stain, Merck Millipore) and digested in gel with trypsin as described previously (3).

LC MS/MS for proteomics

Peptides were desalted with C_{18} StageTips (4) and analysed on a VanquishNeo nano-UHPLC system coupled to an Orbitrap Exploris 480 mass spectrometer via nanoelectrospray ion source (all Thermo Scientific) as described previously (5) with slight modifications: peptides were separated on a 20 cm long analytical column with an inner diameter of 75 µm (CoAnn Technologies), in-house packed with ReproSilPur C18-AQ 1.9µm beads (Dr. Maisch GmbH) using a 49 min segmented gradient of 4-20-35-55% HPLC solvent B (80% acetonitrile in 0.1% formic acid) at a flow rate of 300 nl/min.
In the mass spectrometer, full MS were acquired at resolution 60k with an automatic gain control (AGC) set to standard and a maximum ion injection time (IT) set to automatic. In each scan cycle, the 20 most intense precursor ions were picked up and fragmented using a normalized collision energy (NCE) of 28. Again, AGC and IT were set to standard and automatic, respectively, and the resolution was defined to 15k. Sequenced precursor masses were excluded from further selection for 30 s.

MS data processing for proteomics

MS data were processed using default parameters of the MaxQuant software with integrated Andromeda search engine (v2.5.0.0) (6, 7). Obtained peak lists were searched against Streptomyces hawaiiensis (all strains, 7,547 entries, downloaded from UniProt on 30th of January 2024) and the sequences of ClpP 1-6, and against *Streptomyces lividans* (all strains, 16,038 entries, downloaded from UniProt on 30th of January 2024) and the sequence of ClpP 6, respectively. In addition, data were searched against 285 commonly observed contaminants.
Peptide, protein and modi (8) on site identifications were filtered with a false discovery rate (FDR) of 0.01, estimated by the target-decoy approach. The iBAQ (Intensity Based Absolute Quantification) and LFQ (Label-Free Quantification) algorithms were enabled, as was the “match between runs” option (9).

References

1. Mazodier P, Petter R, Thompson C. 1989. Intergeneric conjugation between *Escherichia coli* and Streptomyces species. J Bacteriol 171:3583-5.
2. Maurizi MR, Clark WP, Katayama Y, Rudikoff S, Pumphrey J, Bowers B, Gottesman S. 1990. Sequence and structure of Clp P, the proteolytic component of the ATP-dependent Clp protease of *Escherichia coli*. J Biol Chem 265:12536-45.
3. Gericke N, Beqaj D, Kronenberger T, Kulik A, Gavriilidou A, Franz-Wachtel M, Schoppmeier U, Harbig T, Rapp J, Grin I, Ziemert N, Link H, Nieselt K, Macek B, Wohlleben W, Stegmann E, Wagner S. 2025. Unveiling the substrate specificity of the ABC transporter Tba and its role in glycopeptide biosynthesis. iScience 28:112135.
4. Rappsilber J, Mann M, Ishihama Y. 2007. Protocol for micro-purification, enrichment, pre-fractionation and storage of peptides for proteomics using StageTips. Nat Protoc 2:1896-906.
5. Nashier P, Samp I, Adler M, Ebner F, Lê LT, Göppel M, Jers C, Mijakovic I, Schwarz S, Macek B. 2024. Deep phosphoproteomics of *Klebsiella pneumoniae* reveals HipA-mediated tolerance to ciprofloxacin. PLoS Pathog 20:e1012759.
6. Cox J, Mann M. 2008. MaxQuant enables high peptide identification rates, individualized p.p.b.-range mass accuracies and proteome-wide protein quantification. Nat Biotechnol 26:1367-72.
7. Cox J, Neuhauser N, Michalski A, Scheltema RA, Olsen JV, Mann M. 2011. Andromeda: a peptide search engine integrated into the MaxQuant environment. J Proteome Res 10:1794-805.
8. Elias JE, Gygi SP. 2007. Target-decoy search strategy for increased confidence in large-scale protein identifications by mass spectrometry. Nat Methods 4:207-14.
9. Tyanova S, Temu T, Cox J. 2016. The MaxQuant computational platform for mass spectrometry-based shotgun proteomics. Nat Protoc 11:2301-2319.
