# Supplementary material for: Self-resistance mechanism to acyldepsipeptide antibiotics in the Streptomyces producer
Source: mBio. 2025 Oct 6;16(11):e01652-25. doi: 10.1128/mbio.01652-25 (PMC12607617; doi:10.1128/mbio.01652-25)
Supplement: Table S1 — Bacterial strains and plasmids. [file mbio.01652-25-s0010.pdf]

# SI file

## Self-resistance mechanism to acyldepsipeptide antibiotics in the *Streptomyces* producer

Dhana Thomy[1,2,4], Laura Reinhardt[1,2,4], Elisa Liebhart[1,2], Mirita Franz-Wachtel[2,3], Boris Maček[2,3], Peter Sass[1,2+], Heike Brötz-Oesterhelt[1,2*+].
[1]Department of Microbial Bioactive Compounds, IMIT, University of Tübingen, Germany. [2]Cluster of Excellence - Controlling Microbes to Fight Infections, University of Tübingen, Germany. [3]Proteome Center Tübingen, University of Tübingen, Germany. [4]Dhana Thomy and Laura Reinhardt contributed equally to this work. Author order was determined by seniority. *heike.broetz-oesterhelt@uni-tuebingen.de.
[+]Peter Sass and Heike Brötz-Oesterhelt share senior authorship.

## Table S1. Bacterial strains and plasmids.

| Strain /plasmid | Relevant characteristic(s)/genotype | Ref. /Source |
|---|---|---|
| *E. coli* | | |
| K-12 JM109 | subcloning host | Thermo Fisher |
| DH5α | subcloning host | Thermo Fisher |
| SG1146a | BL21(DE3) *clpP::cam* | (1) |
| ET12567 pUB307 | *F-dam-13::Tn9 dcm-6 hsdM hsdR zjj-202::Tn10 recF143 galK2 galT22 ara-14 lacY1 xyl-5 leuB6 thi-1 tonA31 rpsL136 hisG4 tsx-78 mtl-1 glnV44; pUB307;* $Cm^R$, $Kan^R$ | (2) |
| *S. lividans* | | |
| TK24 | *str-6;* SLP2$^-$, SLP3$^-$ | DSMZ culture collection |
| Δ*clpP1clpP2* | *str-6;* SLP2$^-$, SLP3$^-$; Δ*clpP1clpP2* | (2) |
| *S. hawaiiensis* NRRL 15010 | wildtype | NRRL culture collection |
| *S. coelicolor* | wildtype | DSMZ culture collection |
| *S. griseus* Waksman | wildtype | DSMZ culture collection |
| pET11a | vector for the expression of native protein expression | Novagen |
| pET22b | vector for the expression of C-terminal His6-fusion protein | Novagen |
| pET11aShclpP1 | pET11a + ORF CEB94_14110 (*S. hawaiiensis clpP1*) | (2) |
| pET11aShclpP2 | pET11a + ORF CEB94_14105 (*S. hawaiiensis clpP2*) | (2) |
| pET22b**Nco*I-ShclpP2-His6 | pET22b**Nco*I + ORF CEB94_14105 (*S. hawaiiensis clpP2*) | (2) |
| pET11aShclgR-N-His6 | pET11a + ORF CEB94_30145 (*S. hawaiiensis clgR*) | (2) |
| pET11aShpopR-N-His-6 | pET11a + MT943519 (*S. hawaiiensis popR*) | (2) |
| pET22b**Nco*I-ShclpX-His6 | pET22b**Nco*I + ORF CEB94_14100 (*S. hawaiiensis clpX*) | (2) |
| pET22b**Nco*I-ShclpC1-His6 | pET22b*NcoI + ORF CEB94_23085 (*S. hawaiiensis clpC1*) | (2) |
| pET22bShclpP2$_{hp}$ | pET22b**Nco*I-ShclpP2$_{ATG2}$-His6 carrying aa mutation S94A, Y96V, Y116V in the *S. hawaiiensis clpP2* gene | (2) |
| pET11aShclpPADEP | pET11a + clpPADEP expressing aa 17-206 | this study |
| pET22bShclpPADEP*-His6 | pET22b + clpPADEP-His6 expressing aa 17-206 | this study |
| pGM-GUS | temperature-sensitive *Streptomyces* shuttle vector *aac(3)IV, oriT,* $P_{ermE}$*_gusA,* $rep_{ts}$ | Günther Muth, Tübingen |

| | | |
|---|---|---|
| pGM-GUS-Xba | based on pGM-GUS, introduction of an XbaI restriction site by site-directed mutagenesis | (2) |
| pGM-GUS-clpP1clpP2 | knockout vector for *S. lividans clpP2clpP2* | (2) |
| pIJ12551 | ΦC31-integrative Streptomyces shuttle vector, protein expression under *ermE** promotor | (2) |
| pIJ12551clpP1 | constitutive protein expression of SlClpP1 | (2) |
| pIJ12551clpP1clpP2 | constitutive protein expression of SlClpP1ClpP2 | (2) |
| pIJ10257 | ΦBT1-integrative Streptomyces shuttle vector, protein expression under *ermE** promotor | (2) |
| pIJ10257clpP2 | constitutive expression of SlClpP2 | (2) |
| pIJ10257clpP2-His | constitutive expression of SlClpP2 with an N-terminal 6xHis-tag | (2) |
| pIJ10257clpP2$_{hp}$ | constitutive protein expression of SlClpP2 with the following mutation(s): S95A, Y97V, Y117V | (2) |
| pIJ6902clpPADEP | inducible expression of ClpPADEP | this study |
| pIJ6902clpPADEP-His | inducible expression of ClpPADEP with C-term. His6 | this study |
| pSET152ermE*ΔHindIII | phiC31-integrativer shuttle-vector | Till Schäberle, Gießen |
| pSET152clpPADEP | constitutive protein expression of ClpPADEP | this study |
| pIJ12551clpPADEP | constitutive protein expression of ClpPADEP | this study |
| pIJ12551clpPADEP-His | constitutive protein expression of ClpPADEP with C-term. His6 | this study |
| pIJ10257clpPADEP | constitutive protein expression of ClpPADEP | this study |
| pIJ10257clpPADEP-His | constitutive protein expression of ClpPADEP with C-term. His6 | this study |

## References


1. Maurizi MR, Clark WP, Katayama Y, Rudikoff S, Pumphrey J, Bowers B, Gottesman S. 1990. Sequence and structure of Clp P, the proteolytic component of the ATP-dependent Clp protease of *Escherichia coli.* J Biol Chem 265:12536-45.
2. Reinhardt L, Thomy D, Lakemeyer M, Westermann LM, Ortega J, Sieber SA, Sass P, Brötz-Oesterhelt H. 2022. Antibiotic Acyldepsipeptides Stimulate the *Streptomyces* Clp-ATPase/ClpP Complex for Accelerated Proteolysis. mBio 13:e0141322.
